# Mathematical model of Na-K-Cl homeostasis in ictal and interictal discharges

**DOI:** 10.1371/journal.pone.0213904

**Published:** 2019-03-15

**Authors:** Anton V. Chizhov, Dmitry V. Amakhin, Aleksey V. Zaitsev

**Affiliations:** 1 Computational Physics Laboratory, Ioffe Institute, Saint Petersburg, Russia; 2 Laboratory of Molecular Mechanisms of Neural Interactions, Sechenov Institute of Evolutionary Physiology and Biochemistry of the Russian Academy of Sciences, Saint Petersburg, Russia; Georgia State University, UNITED STATES

## Abstract

Despite big experimental data on the phenomena and mechanisms of the generation of ictal and interictal discharges (IDs and IIDs), mathematical models that can describe the synaptic interactions of neurons and the ionic dynamics in biophysical detail are not well-established. Based on experimental recordings of combined hippocampal-entorhinal cortex slices from rats in a high-potassium and a low-magnesium solution containing 4-aminopyridine as well as previous observations of similar experimental models, this type of mathematical model has been developed. The model describes neuronal excitation through the application of the conductance-based refractory density approach for three neuronal populations: two populations of glutamatergic neurons with hyperpolarizing and depolarizing GABAergic synapses and one GABAergic population. The ionic dynamics account for the contributions of voltage-gated and synaptic channels, active and passive transporters, and diffusion. The relatively slow dynamics of potassium, chloride, and sodium ion concentrations determine the transitions from pure GABAergic IIDs to IDs and GABA-glutamatergic IIDs. The model reproduces different types of IIDs, including those initiated by interneurons; repetitive IDs; tonic and bursting modes of an ID composed of clustered IID-like events. The simulations revealed contributions from different ionic channels to the ion concentration dynamics before and during ID generation. The proposed model is a step forward to an optimal mathematical description of the mechanisms of epileptic discharges.

## 1. Introduction

Although there are numerous studies on epilepsy, the mathematical modeling of epileptic discharges is still a challenge. The role of nonsynaptic mechanisms in epilepsy is not commonly recognized [1]. Indeed, a large number of mathematical models of epileptic activity that arises from synaptically coupled networks have been developed. In contrast, the number of computational models that take nonsynaptic mechanisms into account is limited. Kager et al. [2–4], Cressman et al. [5], Ullah et al. [6], and Owen et al. [7] used a model of a single neuron embedded in its environment to investigate how changing ionic concentrations in the extracellular space influence neuronal excitability. These models provide valuable insight into the role of local fluctuations of ion concentrations at the single neuron level. Pathological network behaviors mediated by changes in extracellular potassium concentration have been investigated in studies by Bazhenov et al. [8,9], Frohlich et al. [10], and Krishnan et al. [11,12]. A study conducted by Wei and Ullah et al. [13–15] to investigate the oxygen concentration related efficiency of the Na^+^/K^+^ pump and glial buffering in a computational model led to a deeper understanding of the relationship between the breakdown of ion concentration homeostasis and various neurological conditions, including epilepsy.

A well-known recent study of epileptic discharges that included network simulations and a detailed description of ionic dynamics was conducted by Krishnan et al. [12]; the simulated discharges relied on the depolarization block of pyramidal cells, which, however, is not common in experiments. In contrast, the model developed by Gentiletti et al. [1] quite realistically reproduced the initiation of an ictal discharge related to neuronal membrane potential and extracellular potassium concentration; however, the regime of repeating ictal discharges and a regime with interictal-like bursts were not achieved. In contrast, the model presented by Wei et al. [14] reproduced repeated ictal discharges in a pair of coupled excitatory and inhibitory neurons with dynamic concentrations of potassium, sodium, and oxygen without the chloride dynamic. In this article, a model that more precisely describes epileptiform discharges in coupled populations of heterogeneous excitatory and inhibitory neurons with dynamic concentrations of potassium, sodium, and chloride ions with the reproduction of repeated ictal discharges is proposed.

In some experimental models of epilepsy, patterns of epileptiform activity are similar. For instance, there are common features in three such models with slice preparations. The first model uses the combined hippocampal-entorhinal cortex slices from rats maintained in high potassium bath solutions containing 4-aminopyridine (4-AP) [16]. The other two models use human tissue slices. The slices were either taken from human subjects with mesial temporal lobe epilepsies and contained the hippocampus, subiculum, and entorhinal cortex with the activation of epileptic events by high potassium, low magnesium solution [17], or they were taken from peritumoral human cortical tissue [18]. Three types of activity patterns were distinguished: two types of short discharges that last a few hundreds of milliseconds and repeat irregularly with a characteristic interval of the order of seconds and one type with ictal discharges (IDs) that last tens of seconds and repeat on a time scale of minutes. The IDs are often composed of distinct but frequent short discharges. The short discharges fall into two different classes with the dominance of a GABA-A-receptor contribution and with a robust glutamatergic component. The former is referred to as IIDs of the first class (IID1s) in this work [16] and as IIDs by Huberfeld et al. [17] and Pallud et al. [18] and preictal spikes in [19]. The latter is referred to as IIDs of the second class (IID2s) in this work [16] and as preictal discharges (PIDs) by Huberfeld et al. [17,18]. Because the short discharges that constitute IDs are similar to those of IIDs, in the present study, the separation on IID1s and IID2s was used assuming that the mechanisms of IID2s and PIDs are similar. In all three papers, the key factor in the initiation of discharge generation is the reversal of the polarity of the GABA-A-mediated currents in some fraction of neuronal populations due to pathological chloride accumulation within neurons. The major difference in the mechanism of the discharges proposed by Pallud et al. [18] in comparison with that of other experiments [16] consists of the separation of the pyramidal cell population in two subpopulations with normal and pathologically depolarized GABA-A reversal potential. Indeed, as shown in [20], the population of pyramidal neurons is heterogeneous with different values of GABA-A reversal potential due to the different levels of KCC2 expression in the cells [21]. The same evidence of different GABA-A reversal potentials was reported by Glykys et al. [22]; however, the authors proposed an alternative explanation for this heterogeneity and showed that local impermeant anions establish the neuronal chloride concentration. Here, the distribution of GABA-A reversal potential across a population of pyramidal neurons was taken into account by splitting the population into two subpopulations with normal and impaired chloride homeostasis. Based on this hypothesis, a model was developed with two subpopulations of normal and pathological glutamatergic neurons and one population of interneurons.

A sound approach for modeling the neuronal population activity of highly synchronized neurons is a population-type model, but it should be quite precise for transient, non-equilibrium regimes, thus belonging to the class of probability density models [23]. Coupling neuronal excitation with ionic dynamics requires an explicit description of voltage-gated ionic currents. This is possible in the frames of the conductance-based refractory density (CBRD) approach [24,25], which is also compatible with two-compartment neuron models that are required to model the extracellular field potential [26]. The CBRD approach without explicit consideration of ionic dynamics has already been used to model IIDs in our recent publications [27,28]. The main factor that led to the different types of IIDs was a depolarized level of GABA-A reversal potential assumed to be constant during each of the regimes. Whereas this assumption is feasible for IIDs, it does not apply to transient regimes and ID generation. In this study, simulations of IDs are achieved by generalizing the model to an explicit description of the ionic dynamics of three neuronal populations. A sound level of reduction of the ionic dynamics model should describe the changes in ionic concentrations that have the most significant effect on reversal potential. According to Nernst equations, these concentrations include extracellular potassium, intracellular sodium, and chloride concentrations that are smaller than their counterparts on the other sides of the membrane. Hence, these three ions are the focus, which is a tradeoff between the more complex [1,12] and more reduced [5,6,8,14] models of other groups.

## 2. Methods

### 2.1 Experiments

Details of the experimental methods were described previously [16,27]. Shortly, the experiments were carried out in 3-week-old Wistar rats. All animal procedures followed the guidelines of the European Community Council Directive 86/609/EEC and were approved by the Animal Care and Use Committee of the Sechenov Institute of Evolutionary Physiology and Biochemistry of the Russian Academy of Sciences. 35 rats were used in this study. Rats were decapitated and their brains removed rapidly. A vibrating microtome (Microm HM 650 V; Microm; Germany) was used to cut horizontal slices 300-μm thick that contained entorhinal cortex and hippocampus. All steps used artificial cerebrospinal fluid (ACSF) with the following composition (in mM): 126 NaCl, 2.5 KCl, 1.25 NaH2PO4, 1 MgSO4, 2 CaCl2, 24 NaHCO3, and 10 dextrose. The ACSF was aerated with carbogen (95% O2/5% CO2). Recordings were made at 30° C. Pyramidal neurons in deep layers of the entorhinal cortex were visualized using a Zeiss Axioscop 2 microscope (Zeiss; Oberkochen, Germany) equipped with differential interference contrast optics and a video camera (PointGrey Grasshopper3 GS3-U3-23S6M-C, FLIR Integrated Imaging Solutions Inc., USA). Patch electrodes (3–5 MΩ) were pulled from borosilicate filamented glass capillaries (WPI; UK) on a P-1000 Micropipette Puller (Sutter Instrument; Novato, CA, USA). For current-clamp recordings, a potassium-gluconate-based intracellular solution was used. The solution had the following composition (in mM): 135 K-gluconate, 10 NaCl, 5 EGTA, 10 HEPES, 4 ATP-Mg, and 0.3 GTP (with pH adjusted to 7.25 with KOH). For voltage-clamp recordings, a solution based on cesium-methane-sulfonate (CsMeS) was used. This solution had the following composition (in mM): 127 CsMeS, 10 NaCl, 5 EGTA, 10 HEPES, 6 QX314, 4 ATP-Mg, and 0.3 GTP (with pH adjusted to 7.25 with CsOH). Whole-cell recordings were performed with two Model 2400 patch-clamp amplifiers (AM-Systems; Sequim, WA, USA), and an NI USB-6343A/D converter (National Instruments; Austin, TX, USA) using WinWCP5 software (SIPBS; Glasgow, UK). The data were filtered at 10 kHz and sampled at 20 kHz. After the formation of the whole-cell configuration, access resistance was less than 15 M and remained stable (30% increase) during the experiments in all cells included. In the combined entorhinal cortex-hippocampal slices, epileptiform activity was induced with the pro-epileptic solution, containing the following (in mM): 120 NaCl, 8.5 KCl, 1.25 NaH2PO4, 0.25 MgSO4, 2 CaCl2, 24 NaHCO3, 10 dextrose, and 0.05 4-AP. The solution was aerated with carbogen (95% O2/5% CO2) throughout the experiment. The flow rate in the perfusion chamber was 5–6 ml/min. The liquid junction potentials were measured as described [29], and the holding potential was compensated offline for voltage-clamp recordings by subtracting 7 mV. The recordings from 54 slices (1–2 slices per rat) were included in analysis.

Field potentials were recorded using glass microelectrodes (0.2–1.0 MOhm) filled with ACSF and placed in deep layers of the entorhinal cortex. Epileptiform activity was registered with a Model 1800 differential amplifier (AM-Systems; Sequim, WA, USA), digitized and recorded to a personal computer using the acquisition card NI USB-6211 (National Instruments; Austin, TX, USA) and the WinWCP5 software.

### 2.2 Modeling

The proposed model consists of two subsystems that describe neuronal excitability and ionic dynamics. Neuronal excitability has been described in a recent work [27], and the equations are given in the S1 Appendix. The ionic dynamics equations were based on previous works [2,5,8,14] and are described in the following section. The effects of spatial propagation or interactions between different areas were not considered due to the experimental finding [27] that the properties of IDs and IIDs in slices with an isolated entorhinal cortex were similar to those of the combined slices, and thus the entorhinal cortex alone is sufficient to generate and to maintain epileptiform activity. It is assumed that the spatial propagation of neuronal activity within this region is not essential.

#### Neuronal excitability equations

Three neuronal populations were considered, including two excitatory populations and one inhibitory (*I*) population. One excitatory population (*E1*) included neurons that were hyperpolarized by GABA-A-mediated synapses, and in the other (*E2*), they were depolarized. The population dynamics are described in terms of the conductance-based refractory density approach [24,25], which is a generalization of the original refractory density approach [30] for single- or two-compartmental [31] Hodgkin-Huxley-like neurons. The approximations for voltage-gated and synaptic ionic channels were taken from [32,33] and [31], respectively. Lognormal distribution of synaptic weights was implemented as in [34]. The synaptic depression was taken into account in accordance with the Markram-Tsodyks model [35] in its rate-based form [36]. To relate the intrinsic variables of neuronal populations to local field potentials (LFP), the model of coupled populations was supplied by the expression for LFP calculation according to [26]. This approach has been applied to simulations of interictal discharges in an assumption of pathologically shifted but constant reversal potentials of GABAergic currents. Here, the approach is generalized to the accounting of three populations and dynamic ionic concentrations. The equations are given in the S1 Appendix.

#### Ionic dynamics equations

**Fig 1:**
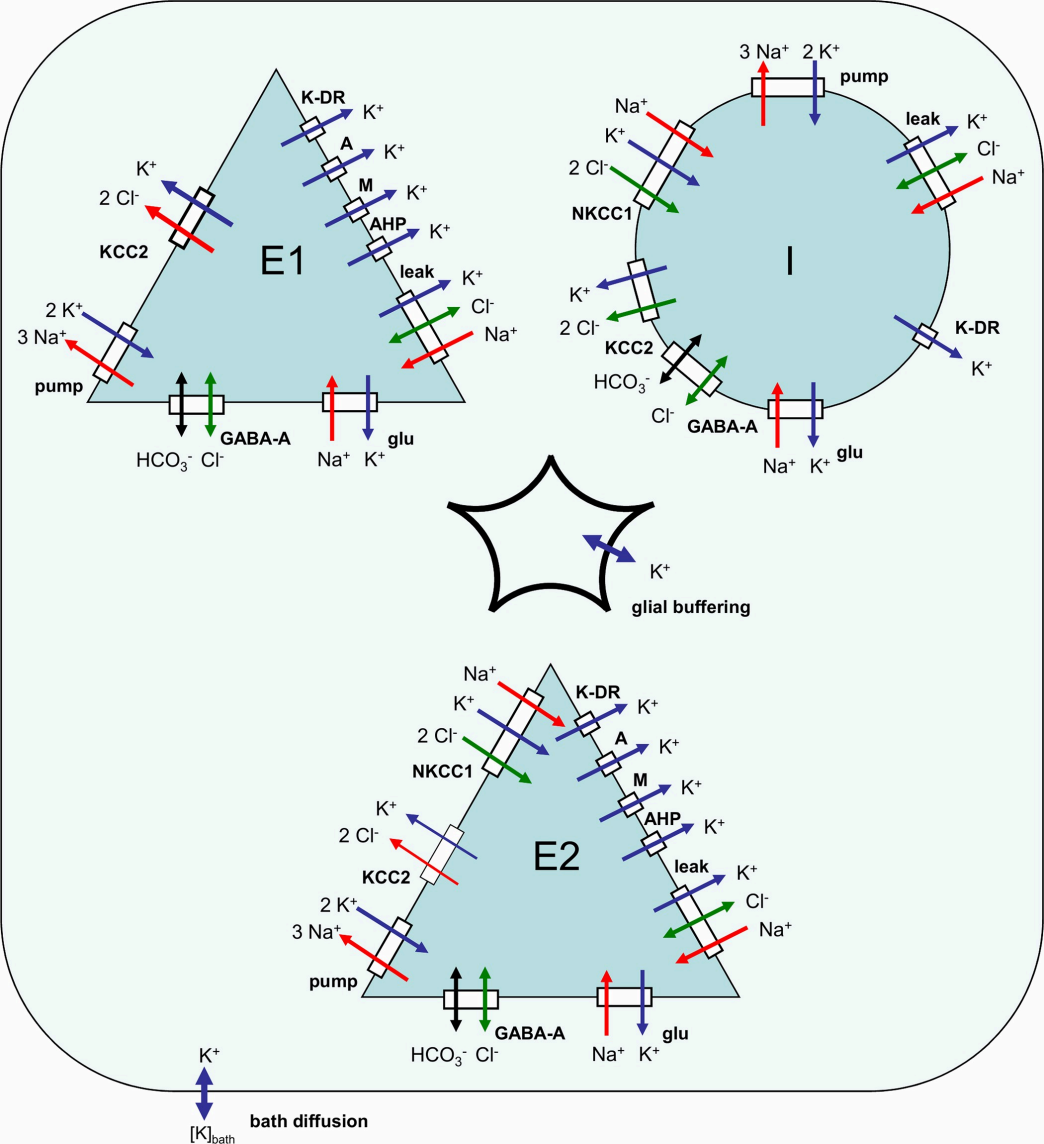
Schematic of ionic dynamics mechanisms. *E1* and *E2* are pyramidal cells with hyper- and depolarizing GABA synapses, correspondingly, *I* is an interneuron. Dynamics of K^+^, Cl^-^, Na^+^ and HCO_3_^-^ ions is determined by the action of the leak, voltage-gated K-DR-, A-, M- and AHP- and synaptic GABA-A- and glutamatergic channels together with the K^+^/Cl^-^ cotransporters KCC2 and NKCC1, and Na^+^/K^+^ exchange pump.

The ionic concentrations that strongly affect reversal potentials are the extracellular potassium concentration [*K*]_*O*_, intracellular chloride concentrations ${\left[Cl\right]}_{i}^{E1}$, ${\left[Cl\right]}_{i}^{I}$, and ${\left[Cl\right]}_{i}^{E2}$ within *E1-*, *E2-* and *i*-neurons, respectively, and the intracellular sodium concentrations ${\left[Na\right]}_{i}^{E1}$, ${\left[Na\right]}_{i}^{I}$, and ${\left[Na\right]}_{i}^{E2}$ (Fig 1).

The potassium balance equation was modified after Wei et al. [14]. The contribution of inhibitory neurons was equal to 1/4 in accordance with the fraction of inhibitory neurons in the cortical tissue [37]. The ratio of *E2*-neurons in the entire pyramidal neuron population is up to 2/3, according to the experimental observations [18]. However a smaller fraction is sufficient for these neurons to play a triggering function.

$$\frac{d{\left[K\right]}_{o}}{dt}=\gamma \beta \left[\begin{array}{l} \alpha_{E1}\left(I_{K,leak}^{E1}+I_{K,active}^{E1}+I_{K,glu}^{E1}-2I_{pump}^{E1}-I_{KCC2}^{E1}\right)+\\ \alpha_{E2}\left(I_{K,leak}^{E2}+I_{K,active}^{E2}+I_{K,glu}^{E2}-2I_{pump}^{E2}-I_{KCC2}^{E2}-I_{NKCC1}^{E2}\right)+\\ \alpha_{I}\left(I_{K,leak}^{I}+I_{K,active}^{I}+I_{K,glu}^{I}-2I_{pump}^{I}-I_{KCC2}^{I}-I_{NKCC1}^{I}\right) \end{array}\right]+G+D\left({\left[K\right]}_{bath}-{\left[K\right]}_{o}\right)$$ (1)

where *β* is the ratio of intra/extracellular volume; *α*_*E*1_,*α*_*E*2_ and *α*_*I*_ are fractions of the numbers of different types of cells in the total number of cells (*α*_*E*1_+*α*_*E*2_+*α*_*I*_ =1); $I_{K,leak}^{E1,E2,I}$ and $I_{K,active}^{E1,E2,I}$ are the potassium currents through leak and active channels, respectively; $I_{K,glu}^{E1,E2,I}$ is the potassium current through glutamatergic channels; $I_{pump}^{E1,E2,I}$ is the current of doublets of the potassium ions; $I_{KCC2}^{E1,E2,I}$ is the flux of potassium due to the KCC2-cotransporter; $I_{NKCC1}^{E2}$ is the potassium flux due to the NKCC1-cotransporter, which is present only in *E2*-neurons; and *γ* is the surface-to-volume and charge-to-concentration translating parameter.

For each of the populations, *E1*, *E2*, and *I*, the intracellular concentrations of the chloride and sodium ions were calculated according to the balance equations:

$$\frac{d{\left[Cl\right]}_{i}}{dt}=\gamma \left(I_{Cl,leak}+I_{GABA}+I_{KCC2}+2I_{NKCC1}\right)$$ (2)

$$\frac{d{\left[Na\right]}_{i}}{dt}=\gamma \left(-I_{Na,leak}-I_{Na,glu}+q_{Na}\nu (t)-3I_{pump}+I_{NKCC1}\right)$$ (3)

where *I*_*Cl*,*leak*_ and *I*_*Na*,*leak*_ are the chloride and sodium leak currents, respectively; *I*_*GABA*_ is the chloride ion current through GABA-A-controlled receptors; and *I*_*Na*,*glu*_ is the sodium current through the glutamatergic receptors. Reversal potentials were obtained from the Nernst equations:

$$V_{K}=26.6mV\;\ln\left({\left[K\right]}_{o}/{\left[K\right]}_{i}\right)$$ (4)

$$V_{Cl}=26.6mV\;\ln\left({\left[Cl\right]}_{i}/{\left[Cl\right]}_{o}\right)$$ (5)

$$V_{Na}=26.6mV\;\ln\left({\left[Na\right]}_{o}/{\left[Na\right]}_{i}\right)$$ (6)

$$V_{GABA}=26.6mV\;\ln\left(\left(4{\left[Cl\right]}_{i}+{\left[HCO_{3}\right]}_{i}\right)/\left(4{\left[Cl\right]}_{o}+{\left[HCO_{3}\right]}_{o}\right)\right)$$ (7)

The Na^+^/K^+^ pump [13,14], KCC2 [1,13,38,39], and NKCC1 [13,39] transporter currents were calculated as follows:

$$I_{pump}=\frac{I_{pump,\max}}{\left(1+\exp(3.5-{\left[K\right]}_{o}))(1+\exp((25-{\left[Na\right]}_{i})/3)\right)}$$ (8)

$$I_{KCC2}=I_{KCC2,\max}\left(V_{K}-V_{Cl}\right)/26.6\;mV$$ (9)

$$I_{NKCC1}=I_{NKCC1,\max}\left(V_{Na}+V_{K}-2V_{Cl}\right)/26.6\;mV$$ (10)

Ionic channel currents were calculated based on the somatic and dendritic membrane potentials averaged across the entire population:

$$\bar{U}(t)=\int_{0}^{\infty }U(t,t^{*})\int_{0}^{\infty }\;\rho_{x}(t,t^{*})\psi (x)dxdt^{*}$$ (11)

and

$$\bar{U_{d}}(t)=\int_{0}^{\infty }U_{d}(t,t^{*})\int_{0}^{\infty }\;\rho_{x}(t,t^{*})\psi (x)dxdt^{*}$$ (12)

with the lognormal distribution taking into account heterogeneity of synaptic weights within a population

$$\psi (x)=\frac{\exp(-{\left(\ln x\right)}^{2}/(2\sigma_{LN}^{2}))}{\sqrt{2\pi }\sigma_{LN}x}.$$ (13)

Eqs (11–13) repeat eqs. (A30,A33,A34). In these equations, the density of neuronal distribution across t*- the time elapsed since the last spike, *ρ*_*x*_(*t*,t*), is calculated with the refractory density equation for different populations, eqs.(A1,A24) given in S1 Appendix; the distribution across t* of the somatic and dendritic membrane potentials *U*(*t*,t*) and *U*_*d*_(*t*,t*) is calculated with eqs. (A2,A3,A25).

The leak currents are as follows:

$$I_{K,leak}=g_{KL}\left(\bar{U}-V_{K}\right),\;I_{Cl,leak}=g_{ClL}\left(\bar{U}-V_{Cl}\right),\;I_{Na,leak}=g_{NaL}\left(\bar{U}-V_{Na}\right).$$ (14)

$$I_{K,active}=\int_{0}^{\infty }\left(I_{K-DR}(t,t^{*})+I_{M}(t,t^{*})+I_{AHP}(t,t^{*})\right)\int_{0}^{\infty }\rho_{x}(t,t^{*})\psi (x)dxdt^{*}$$ (15)

is the average potassium current through the voltage-gated channels. The M- and AHP-components are absent for interneurons.

The glial buffer is:

$$G=k_{1}(B_{\max}-B)/k_{1N}-k_{2}B,$$ (16)

$$\frac{dB}{dt}=k_{1}(B_{\max}-B)-k_{2}B,\;k_{2}=k_{1}/\left(1+\exp\left(-({\left[K\right]}_{o}-15mM)/1.15mM\right)\right).$$ (17)

The chloride current through the GABAergic channels is:

$$I_{GABA}^{}=g_{GABA}^{}({\bar{U}}_{d}^{}-V_{Cl}^{})$$ (18)

The potassium and sodium currents through the glutamatergic channels [40] were approximated as linear dependent on voltage with the fractions of the total glutamatergic conductance. The fraction was estimated based on the data provided by Mayer and Westbrook [41] as 0.2 for the potassium and 0.4 for the sodium ions. At the level of -50mV, this ratio provides compensation of potassium by calcium currents through the glutamatergic receptor and by the equality of the sodium current to the total glutamatergic current. Thus, the potassium and sodium components were assumed to be:

$$I_{K,glu}^{}=0.2g_{glu}^{}({\bar{U}}_{d}-V_{K}),\;I_{Na,glu}^{}=0.4g_{glu}^{}({\bar{U}}_{d}-V_{Na}).$$ (19)

The ***parameters*** are as follows:

*γ* = *S*/(*F vol*) = 2⋅10^−5^ [(*mM*/*ms*)/(*μA*/*cm*^2^)] (close to the estimate by Barreta and Cressman [42], which is 4.4⋅10^−5^ there); *β* = 5; *α*_*E*2_ = 0.1, *α*_*I*_ = 0.25 (i.e., *α*_*E*1_ = 0.65). The Na^+^/K^+^ pump, KCC2 and NKCC1 transporter current amplitudes are as follows: *I*_*pump*,max_ = 3*μA*/*cm*^2^, $I_{KCC2,\max}^{E1}=2\mu A/cm^{2}$, $I_{KCC2,\max}^{E2}=I_{KCC2,\max}^{I}=0.5\mu A/cm^{2}$, $I_{NKCC1,\max}^{E1}=0$, $I_{NKCC1,\max}^{E2}=I_{NKCC1,\max}^{I}=0.1\mu A/cm^{2}$, [*Cl*]_*o*_ = 130*mM*, [*K*]_*i*_ = 129*mM*, [*Na*]_*o*_ = 130*mM*, [*HCO*_3_]_*o*_ = 24*mM*, and [*HCO*_3_]_*i*_ = 16*mM*. The leak conductances are the same for all three populations: *g*_*KL*_ = 50*μS*/*cm*^2^, *g*_*ClL*_ = 10*μS*/*cm*^2^, and *g*_*NaL*_ = 5*μS*/*cm*^2^. The membrane area is 10^−4^*cm*^2^. The sodium charge transferred by a single spike is *q*_*Na*_ = 0.3*μC*/*cm*^2^. The initial concentrations are [*K*]_*o*_ = [*K*]_*bath*_, ${\left[Cl\right]}_{i}^{E1}=6mM$, ${\left[Cl\right]}_{i}^{E2}={\left[Cl\right]}_{i}^{I}=19mM$, and ${\left[Na\right]}_{i}^{E1}={\left[Na\right]}_{i}^{E2}={\left[Na\right]}_{i}^{I}=12\;mM$. The glial pump was neglected in all simulations except Simulation 4, which was set as *k*_1_ = 0.01*s*^−1^.

The concentrations of intracellular potassium and extracellular chloride and sodium ions are assumed to be constant because their effect on the network is much smaller than that for their counterparts. The sensitivity of the network to changes of the concentrations can be evaluated with the absolute values of the derivatives of the reversal potentials (Eqs (4–6)) on the concentrations, i.e. |∂*V*_*ion*_/∂[*ion*]| = 26.6*mV*/[*ion*]. With the parameters set above and [*K*]_*bath*_ = 8.5*mM*, we obtain |∂*V*_*K*_/∂[*K*]_*i*_| = 0.2*mV*/*mM*, |∂*V*_*Cl*_/∂[*Cl*]_*o*_| = 0.2*mV*/*mM* and |∂*V*_*Na*_/∂[*Na*]_*o*_| = 0.2*mV*/*mM*, which are about one order of magnitude smaller than |∂*V*_*K*_/∂[*K*]_*o*_| = 3.1*mV*/*mM*, |∂*V*_*Cl*_/∂[*Cl*]_*i*_| = 4.4*mV*/*mM* and |∂*V*_*Na*_/∂[*Na*]_*i*_| = 2.2*mV*/*mM*, correspondingly.

## 3. Results

### 3.1 Experimental observations of ictal and interictal discharges

**Fig 2:**
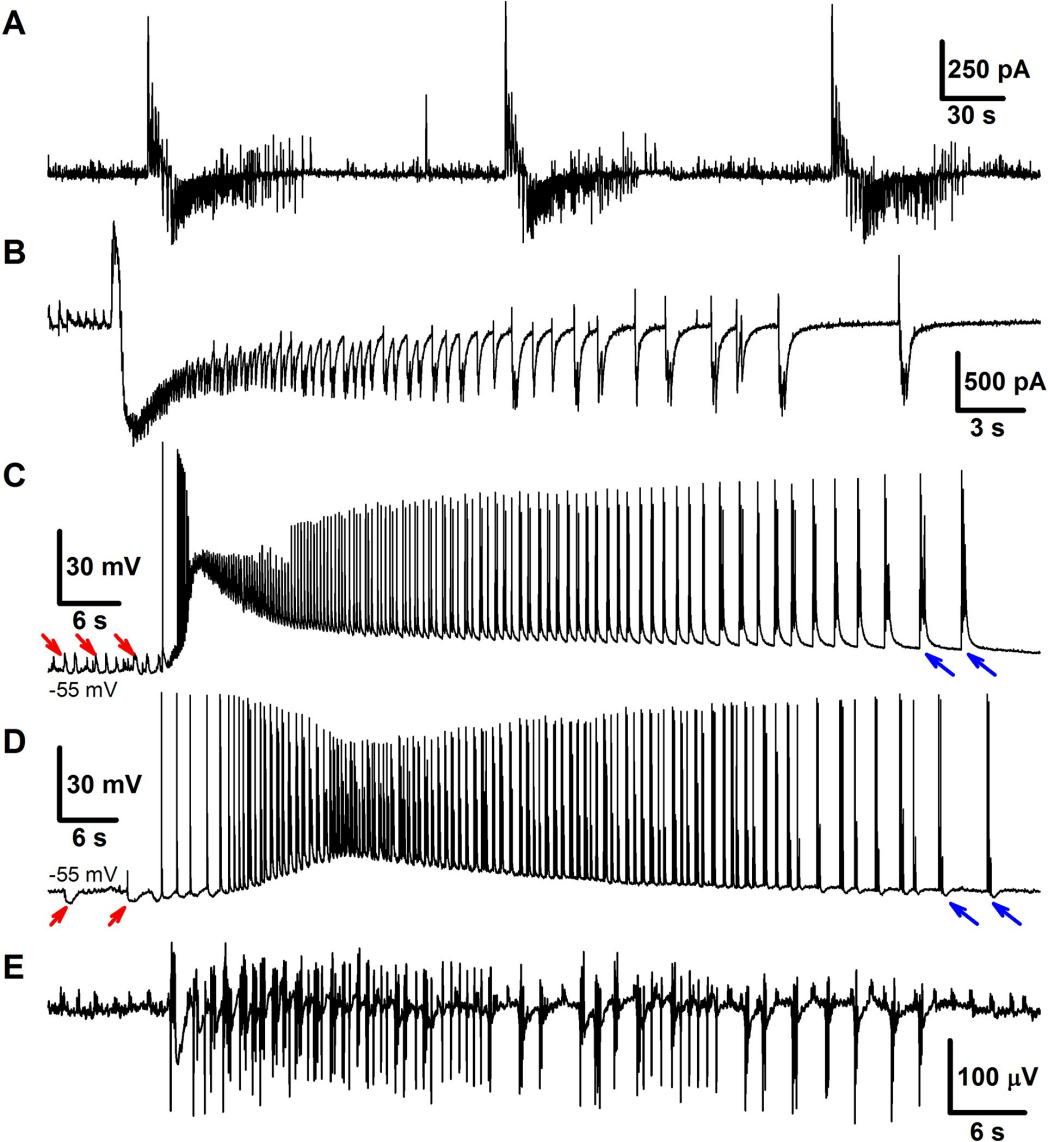
Experiment. **Ictal discharges in combined entorhinal cortex-hippocampal slices. A, B**: IDs recorded in the voltage-clamp mode at *V*_*hold*_ = −27*mV*. **A**: Sequence of three IDs. **B**: Another example of a single ID. **C, D**: IDs recorded in the current-clamp mode. Presumably, the trace in C is from a pyramidal cell with depolarizing GABA-A synapses (>90% of recordings in our preparation), whereas in D from a pyramidal cell with hyperpolarizing GABA-A synapses (less than 10% of the recordings). Red arrows point on several GABA-mediated discharges, that precede ID and are either depolarizing (C), or hyperpolarizing (D). Blue arrows point on discharges that constitute the late phase of ID and have a strong GABA-mediated component which is either depolarizing (C), or hyperpolarizing (D). E: An example of extracellular field recoding during ID.

As reported previously [16,27], following the perfusion of brain slices containing the entorhinal cortex and hippocampus with a pro-epileptic solution, IDs and two types of IIDs, IID1s, and IID2s, were observed. In our preparation, 67% of slices included in analysis were able to generate IDs within the recording period of 30 minutes. Representative examples of IDs recorded in voltage clamp (VC) mode at a holding voltage of -27 mV and in current-clamp (CC) mode in entorhinal pyramidal neurons are shown in Fig 2.

The IDs repeated with an interval of a few minutes (the average interval was 185±10s (n = 17 slices)) (Fig 2A). For the current-clamp recordings, the activity was characterized by action potential burst discharges lasting 30–80 s (the average ID duration was 96±8s (n = 20 slices)) (Fig 2C and 2D). Recordings in voltage-clamp mode at a holding voltage of -27 mV revealed inhibitory and excitatory components of synaptic currents. For this membrane potential, the GABAergic current flows outward, whereas the glutamatergic AMPAR- and NMDAR-mediated currents flow inward. All IDs began with either a short burst of high-amplitude IPSCs or a prolonged burst of IPSCs with lower amplitude (Fig 2A and 2B). The main portion of each ID was composed of stereotypical bursts of currents with components that reversed at various voltages [16]. This stereotypical current burst lasted about 1 s and had the fast IPSCs initially followed by overlapping EPSCs. In most cases, these stereotypical current bursts were clustered at the onset of an ID, forming the “tonic” phase of the ictal event. At a later stage, these current bursts emerged at lower frequencies and formed the “clonic” phase of an ID.

**Fig 3:**
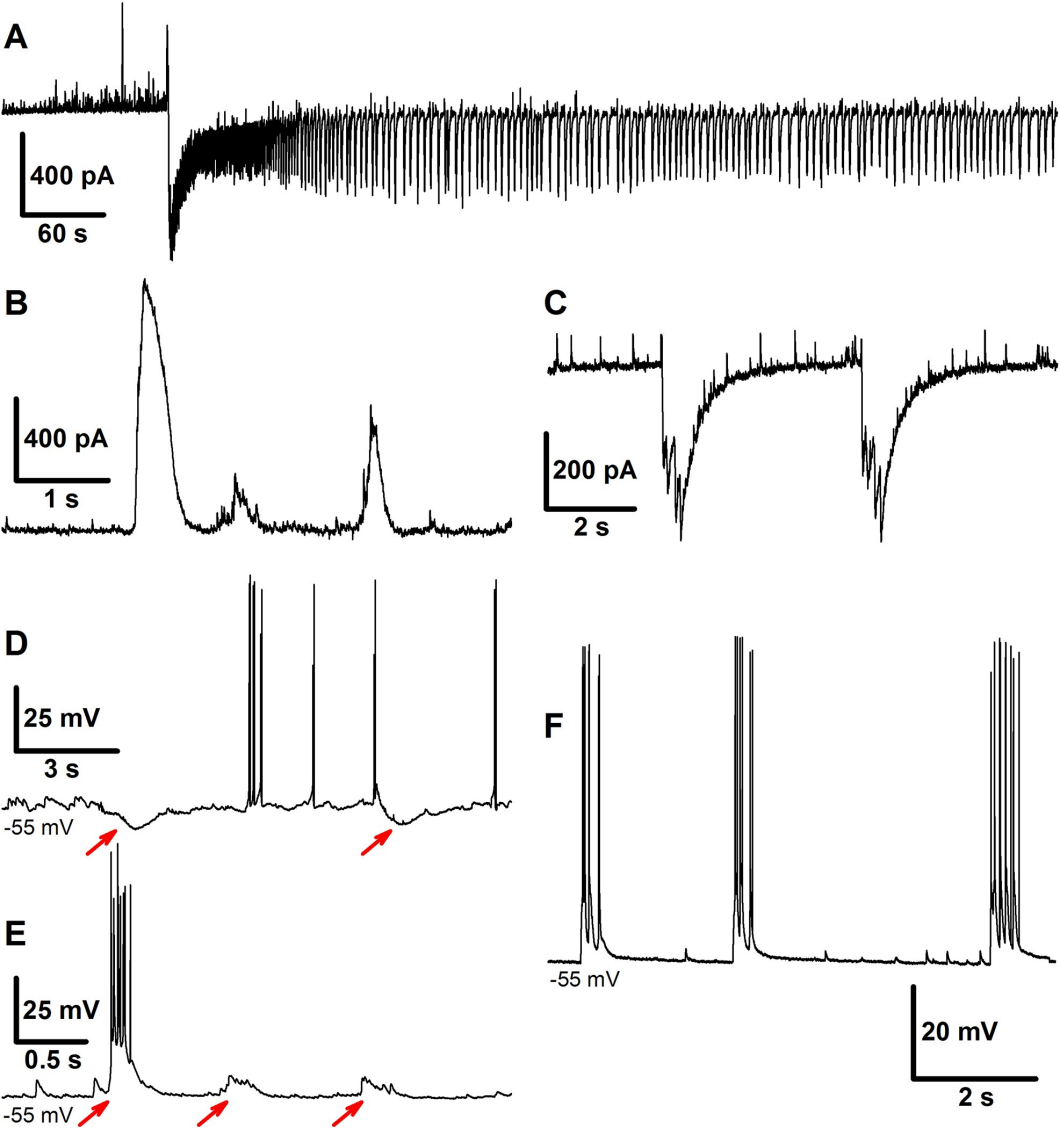
Experiment. **Interictal discharges. A**: Transition from IID1s through ID to IID2s recorded in the voltage-clamp mode. **B**: An example of a few IID1 in VC. **C**: An example of a few IID2s in VC. **D**: Two IID1s exposed as hyperpolarizing events between spontaneous spikes in CC. IID1s are marked with red arrows. **E**: Three IID1s exposed as depolarizing events in CC. IID1s are marked with red arrows. **F**: Three IID2s recorded in CC.

An example of the activity, which is shown in Fig 3A, illustrates a transition from IID1s via an ID to a continuous mode of IID2 generation. In our preparation, 22% of slices exhibited this form of epileptiform activity, which constitutes 33% of those able to generate IDs (for the rest, the generation of IDs persisted more than 30 minutes). In a recent work [16], IID1s and IID2s were classified, and GABA, AMPA, and NMDA synaptic components were identified for each type of discharge. IID1s are pure GABAergic events that reflect a synchronized activity of interneurons under the conditions of depolarized GABA reversal potential. They have observed as purely outward (positive) currents in the recordings at a holding voltage of -27 mV (Fig 3B). IID2 is a sequence of GABAergic and glutamatergic components. IID2s have both positive and negative components in this recording (Fig 3C). A typical duration of IIDs is about 1 s, and the inter-event interval is about a few seconds. The amplitudes and durations of IID1s, as well as the inter-burst interval, are more variable for IID1s than for IID2s. The exact parameters of the time course of synaptic conductances during both types of IIDs in our preparation were reported in our previous works [16,27].

In the present study, the heterogeneity of a population of pyramidal cells was introduced into the model in order to simulate the epileptiform activity. In our preparation, during IIDs the GABA-A conductance was either depolarizing (Figs 2C and 3E) or hyperpolarizing (Figs 2D and 3D), though the latter case was observed in less than 10% of cells. Intracellular chloride accumulation is induced by the pro-epileptic solution. We speculate that in some of the cells, it is perfectly balanced by its extrusion with KCC2-cotransporters and due to equilibration with pipette solution; while in others, it is not. The chloride level changes during IDs [22]. Thus, the effect of GABA may reverse during a single ID. Due to this heterogeneity, the interneurons play a dual role by triggering and terminating the discharges, which makes it necessary to consider at least two subpopulations of pyramidal neurons in a modeling study of epileptic activity observed under these and similar conditions [18].

### 3.2 Simulations

#### Silent state under normal conditions

Under normal conditions, ([*Mg*]_*o*_ = 2 *mM* and [*K*]_*bath*_ = 3.5 *mM*), the system is silent with the steady state variables *U*^*E*1^ = −75*mV*, *U*^*E*2^ = −70*mV*, *U*^*I*^ = −70*mV*, *ν*^*E*1^ = *ν*^*E*2^ = *ν*^*I*^ = 0, [*K*]_*o*_ = 5*mM*, ${\left[Cl\right]}_{i}^{E1}={\left[Cl\right]}_{i}^{I}=6mM,$ ${\left[Cl\right]}_{i}^{E2}=18mM,$ ${\left[Na\right]}_{i}^{E1}={\left[Na\right]}_{i}^{I}=14mM,$ and ${\left[Na\right]}_{i}^{E2}=15mM$. This trivial simulation confirms the consistency of the model with the experimental observations that show no spontaneous activity in the slices immersed in the low-magnesium and low-potassium solution.

#### Types of spontaneous discharges under pro-epileptic conditions

**Fig 4:**
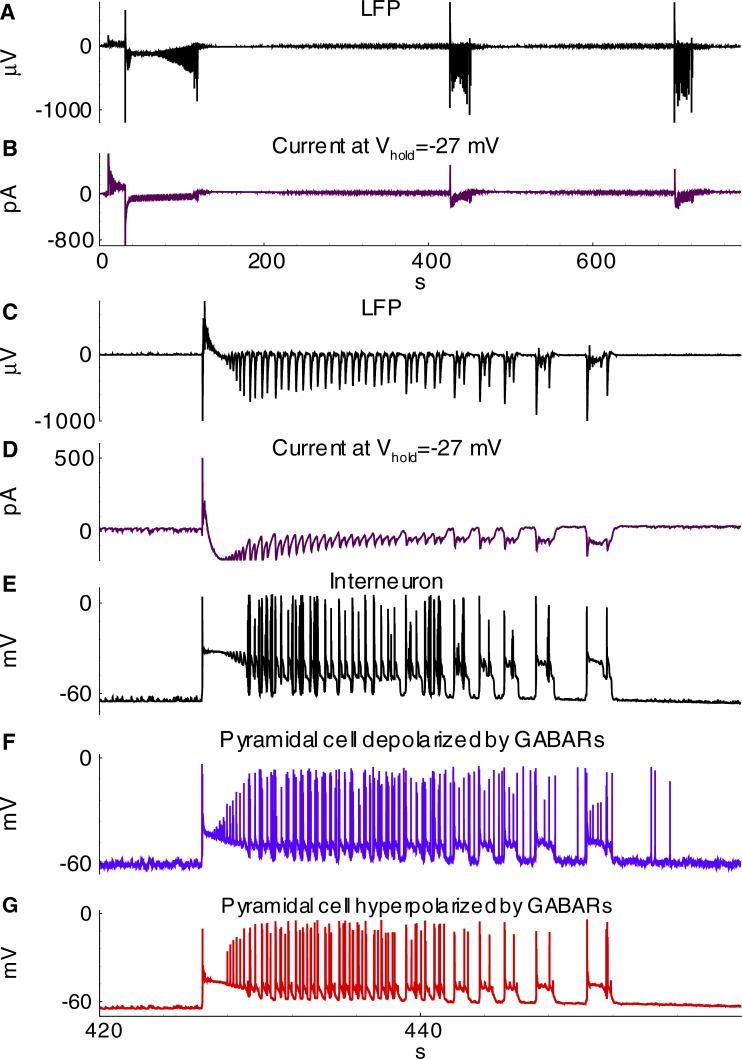
Simulated epileptiform activity with ictal and interictal discharges (Simulation 1). **A**, Local field potential (LFP); **B**, Current recorded in a pyramidal cell (population E1) at the hold voltage -27mV; **C**-**G**, Zoomed interval; **C**, LFP; **D**, current at -27 mV in a cell of population *E1*; **E-G**, membrane potential of cells representing interneurons (population *I*), pyramidal cells depolarized by GABARs (population *E2*) and pyramidal cells hyperpolarized by GABARs (population *E1*).

**Fig 5:**
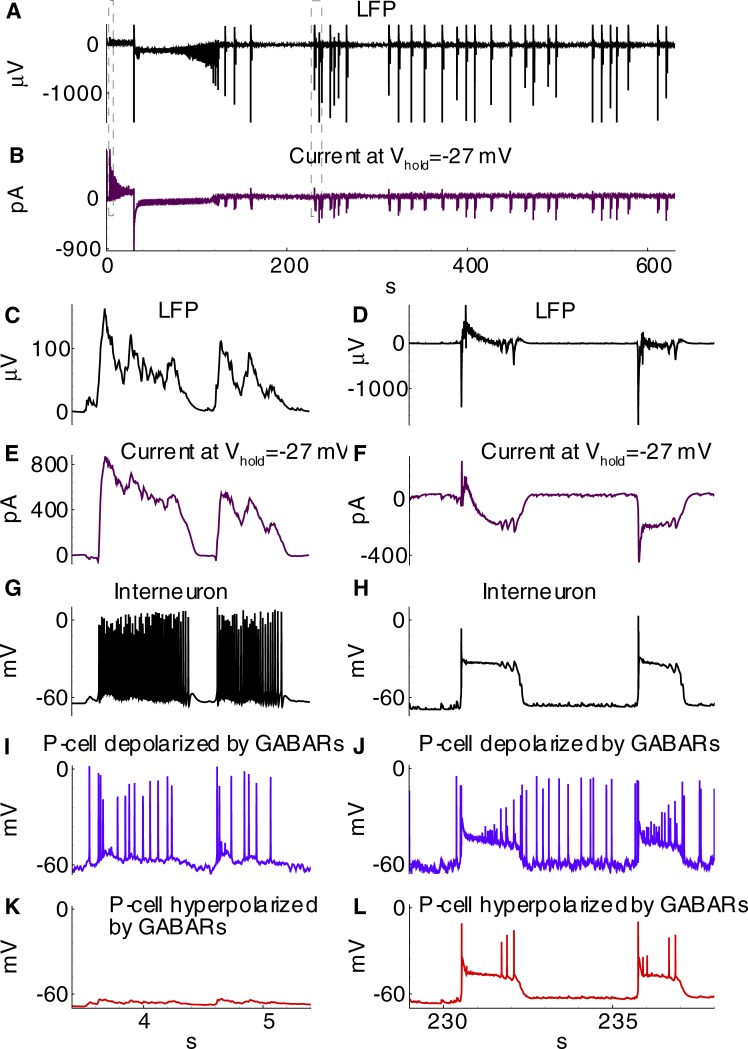
Simulated interictal discharge regime (Simulation 2). **A**, Local field potential (LFP); **B**, Current recorded in a pyramidal cell (population E1) at the hold voltage -27mV; **C**-**L**, Zoomed interval with IIDs of different types, IID1 (left panels) and IID2 (right panels); **C,D**, LFP; **E,F**, current at -27 mV in a cell of population 1; **G-L**, membrane potential of cells representing interneurons (population I), pyramidal cells depolarized by GABARs (population E2) and pyramidal cells hyperpolarized by GABARs (population E1).

The model showed IDs and IIDs. Epileptic events (Figs 4 and 5) were obtained by setting [*Mg*]_*o*_ = 0.25*mM*, affecting NMDARs via eq.(A36) (S1 Appendix), and [*K*]_*bath*_ = 8.5 *mM* affecting [*K*]_*o*_ via Eq (1). A regime with regular IDs (Fig 4) was obtained with relatively low noise *I*_*noise*_ present in eq.(A2) for *E2-*cells (20 pA). With a larger amplitude (40 pA), an ID turns to a regime of the stable generation of IIDs (Fig 5A and 5B), similar to the scenario of the experimental recordings (Fig 3A). The discharges were similar to those observed in experimental models with hippocampal-entorhinal cortex slices in high-potassium bath solutions containing 4-AP, as described in Section 3.1 and previous studies [16–18]. In particular, the pattern of the extracellular potential during ID (Fig 2C) is similar to those recorded in [43,44] and our experiment (Fig 2E); an ID is composed of separate IID-like bursts, each starting from sharp positive-negative and continuing with mostly negative components. The IDs repeat on a time scale of minutes (Fig 4, compare to Fig 2), whereas IIDs are irregular with a characteristic interval of the order of a few seconds (Fig 5, compare to Fig 3).

Activities involving IIDs include IIDs that are GABA-A-component dominated (IID1) and those with both GABAergic and glutamatergic components (IID2) (Fig 5). IID1s are recorded as pure positive pulses in the VC mode of patch-clamp registrations (Fig 5E). They are not quite visible in the CC mode for *E1*-cells (Fig 5K). Such events are similar to experimentally observed IID1s shown in Fig 3B, 3D, and 3E, which are reported here [16], and IIDs from the works by Pallud et al. [18] and Huberfeld et al. [17]. Specifically, the current pulses in Fig 5E correspond to those in Fig 3B; spiking and depolarization during IID1s can be observed in Fig 5I and Fig 3E, and the silence of the E2-cell during IID1s (Fig 5K) is consistent with the silent periods during hyperpolarization (Fig 3D).

The other type of short discharges shown in Fig 5D, 5F, 5H, 5J, and 5L is composed of both GABA and glutamatergic components. In the recordings simulated for the VC mode at -27 mV, they often have small positive, GABAergic and large negative, glutamatergic components (Fig 5F). These events are similar to IID2 recordings reported by Amakhin et al. [16] and Huberfeld et al. [17,18]. Precisely, the current pulses in Fig 5F correspond to those in Fig 3C, and spiking and depolarization during IID2s can be observed for both subpopulations of pyramidal cells (Fig 5J and 5L) and are similar to the experimental patterns in Fig 3F.

#### Mechanism of IID1s

**Fig 6:**
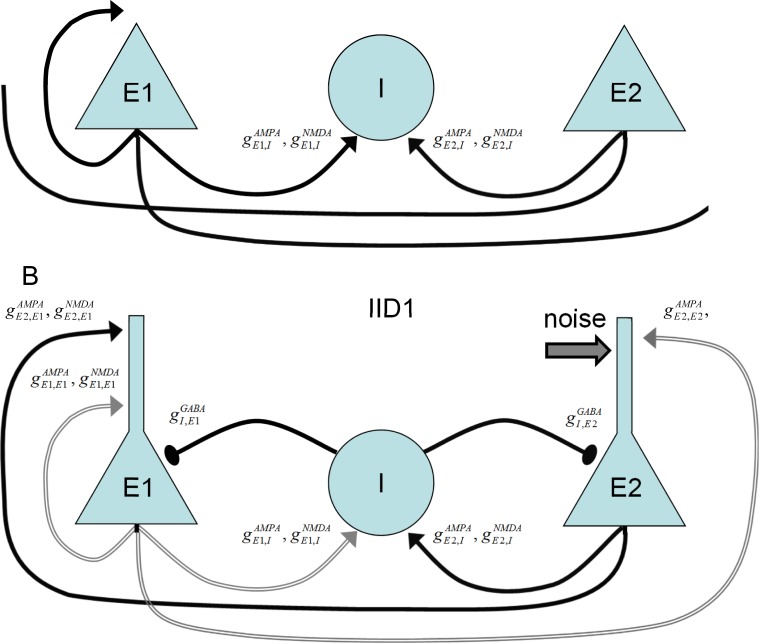
The schematic of spontaneous discharge generation includes the populations of the glutamatergic neurons with hyperpolarizing GABA-A synapses (*E1*), the GABA-ergic interneurons (*I*) and the glutamatergic neurons with depolarizing GABA-A synapses (*E2*). **A**, All the synaptic pathways are involved in the generation of IDs and IID2s. **B**, Only two populations *I* and *E2* contribute to the generation of IID1s.

**Fig 7:**
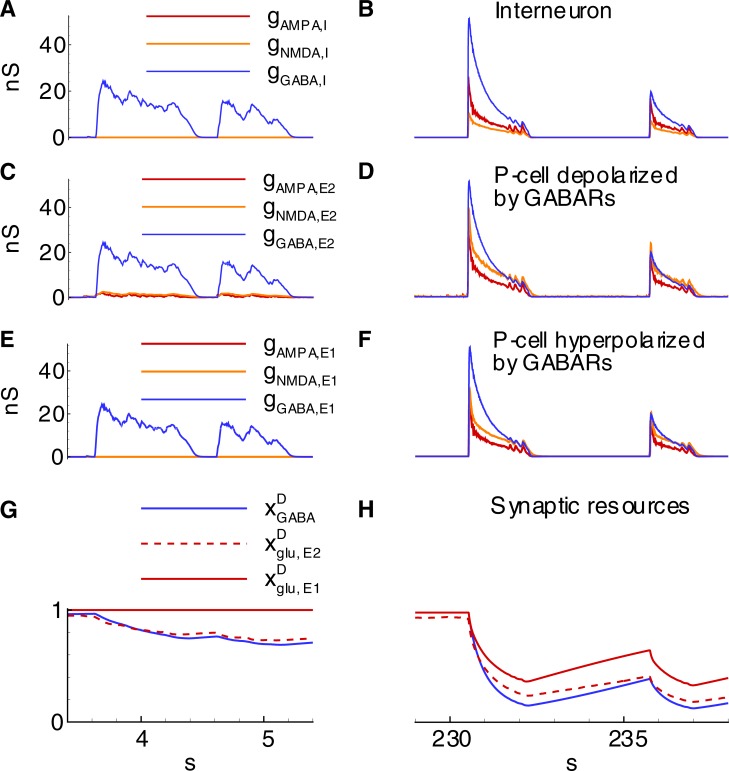
Synaptic conductances during IIDs corresponding to those in Fig 5 (Simulation 2). **A-F**, Synaptic conductances during IID1s (A,C,E) and IID2s (B,D,F) in *I-* (A,B), *E2-* (C,D) and *E1-*type (E,F) neurons. **G, H**, Synaptic resource dynamics during IID1s (G) and IID2s (H).

The mechanisms of the observed IID1s, IID2s, and IDs involve three populations, as reported by Pallud et al. [18], which include glutamatergic neurons with hyperpolarizing GABA-A synapses (*E1*), GABAergic interneurons (*I*), and glutamatergic neurons with depolarizing GABA-A synapses (*E2*) (Fig 6); however, only two populations are active during IID1s. The glutamatergic neurons with depolarizing GABA-A synapses (*E2*) are driven by noise. They generate rare spikes (for instance, see the first spike in Fig 5I). These rare spikes excite interneurons (Fig 5G). The interneurons then depolarize the *E2*-neurons (Fig 5I) and amplify their spike generation, thus providing positive feedback. Consequently, *I*-cell firing activity becomes strong, and the activity of *E2*-neurons is significant, though not sufficient to excite the *E1*-population (Fig 5K). The concentration of chloride in these neurons is low, and GABA-A receptors provide hyperpolarization. *E1*-neurons remain silent. The termination of an IID1 occurs after the weakening of GABA-A receptors (Fig 7A, 7C and 7E) due to a synaptic depression, which is characterized by the synaptic resource variable *x*^*D*^_*GABA*_ (Fig 7G). Overall, the activities of only two populations, *I* and *E2*, determine the generation of IID1s.

**Fig 8:**
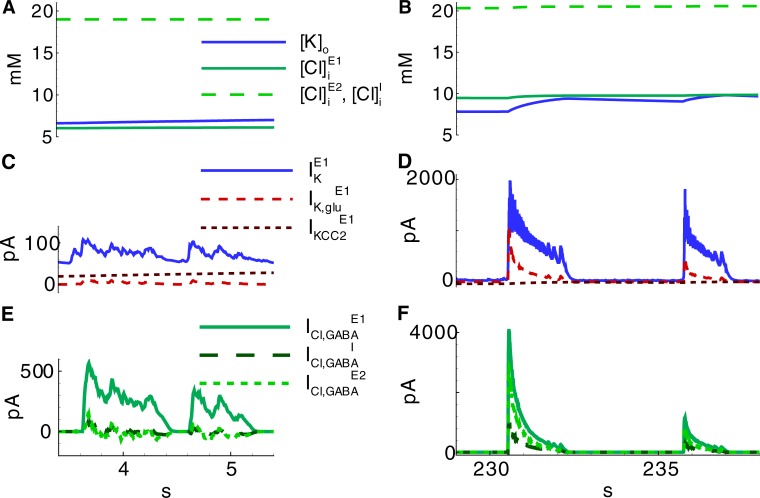
Ionic currents and concentrations during IIDs corresponding to those in Fig 5 (Simulation 2). **A, B**, Extracellular potassium and intracellular chloride ion concentrations during IID1s (A) and IID2s (B). **C, D**, Potassium currents through voltage-gated and glutamatergic channels, and KCC2-cotransporters for *E1-*neurons during IID1s (C) and IID2s (D). **E, F**, Chloride currents through GABAergic channels for *E1-*, *I-* and *E2*-neurons during IID1s (E) and IID2s (F).

The critical factor in the generation of IID1s is the depolarization of the *E2*-neurons by GABA-A receptors. The positive driving force of these receptors is explained by a high level of chloride within *E2*-neurons (${\left[Cl\right]}_{i}^{E2}$ in Fig 8A). According to Pallud et al. [18], this level is provided by the activity of overexpressed NKCC1 cotransporters in this set of neurons, which are included in the proposed model. Consequently, the reversal potential of GABA-A receptors is close to the threshold of spike generation in *E2*-neurons, whereas it is hyperpolarized in *E1*-neurons. Thus, *I*-cells excite *E2*-neurons, providing positive feedback to their triggering activity. On the contrary, *E1*-neurons are inhibited by *I*-cells.

**Fig 9:**
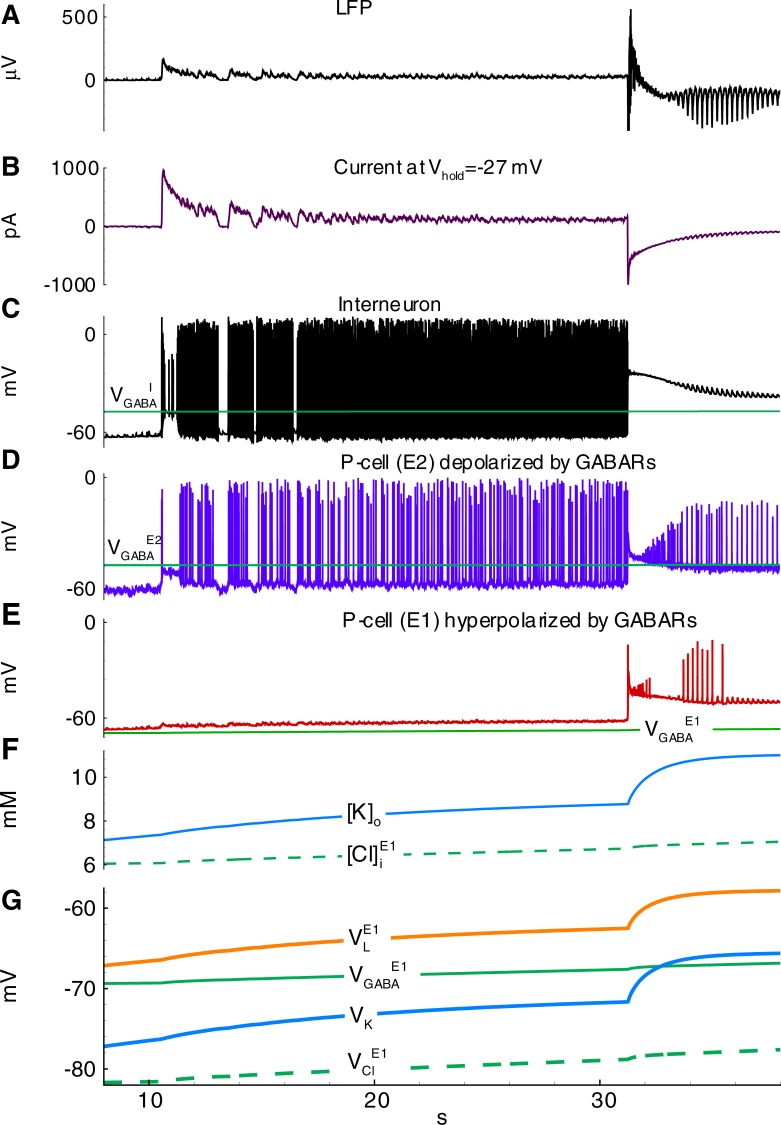
Initiation of the first ID in series of IDs shown in Fig 4 (Simulation 1). **A**, LFP; **B**, current at -27 mV in a cell of population *E1*; **C-E**, the membrane potential and GABA reversal potential for the cells representing interneurons (population I), pyramidal cells depolarized by GABARs (population E2) and pyramidal cells hyperpolarized by GABARs (population E1). **F**, Extracellular potassium and intracellular (in *E1*-neurons) chloride ion concentrations. **G**, Reversal potentials of the leak, GABAergic, chloride leak in *E1*-neurons, and potassium currents.

The ionic concentration levels begin to change after the generation of IID1s (${\left[K\right]}_{o},{\left[Cl\right]}_{i}^{E1}$ in Fig 8A). IID1 generation involves active chloride transport through GABA-A receptors (Fig 8E). While under normal conditions any increase of ${\left[Cl\right]}_{i}^{E1}$ is compensated by slow pumping through KCC2-cotransporters, for the regime of IID1 generation, chloride homeostasis is impaired. The flux of chloride during IID1s overcomes the KCC2 outflux if IID1s become frequent, and the concentration ${\left[Cl\right]}_{i}^{E1}$ gradually increases (Fig 8A). The chloride concentrations in *E2-* and *I*-cells remain constant (Fig 8A) due to the balance of the outflow through KCC2 and the influx through GABA-A receptors. It should also be noted that prominent GABAergic shunting leads to equalization in membrane voltage to *V*_*GABA*_; however, the influx of chloride ions is supported by a more negative level of *V*_*Cl*_ in comparison with *V*_*GABA*_ (Fig 9G) due to the contribution of the HCO_3_ ions according to Eq (7).

The concentration [*K*]_*o*_ increases due to the potassium outflux through KCC2-cotransporters, the voltage-gated channels of active *I-* and *E2*-cells, and the glutamatergic receptors presynaptically stimulated by *E2*-neurons (Fig 8C). As the largest population, the population of *E1*-cells provides the most significant contribution to the change in [*K*]_*o*_. The increase in [*K*]_*o*_ from burst to burst (Fig 9F) provides a gradual increase in depolarization by affecting the reversal potentials of the leak and voltage-dependent potassium channels (Fig 9G).

#### Mechanism of IID2s

Both GABAergic and glutamatergic synapses contribute to IID2s, as evident based on the profiles of the AMPA, NMDA, and GABA-A-conductances (Fig 7B, 7D and 7F), which are similar to those estimated in the experiments [16]. The presynaptic resources of neurotransmitters are partially exhausted at the end of each IID2 (Fig 7H). The weakening of the synapses results in the termination of the IID2. The ionic concentrations of extracellular potassium and intracellular chloride increase during each IID2 and relax during the interburst intervals (Fig 8B). The increase in [*K*]_*o*_ is induced by the voltage-gated and glutamatergic potassium currents together with the flux through KCC2 cotransporters (Fig 8D). The contribution of KCC2 cotransporters is relatively small during the discharges. However, it is effective on large time scales of inter-discharge intervals, after being triggered by the enhanced level of intracellular chloride. The influx of chloride into all types of cells is supplied by the GABAergic channels (Fig 8F).

#### Mechanism of IDs

**Fig 10:**
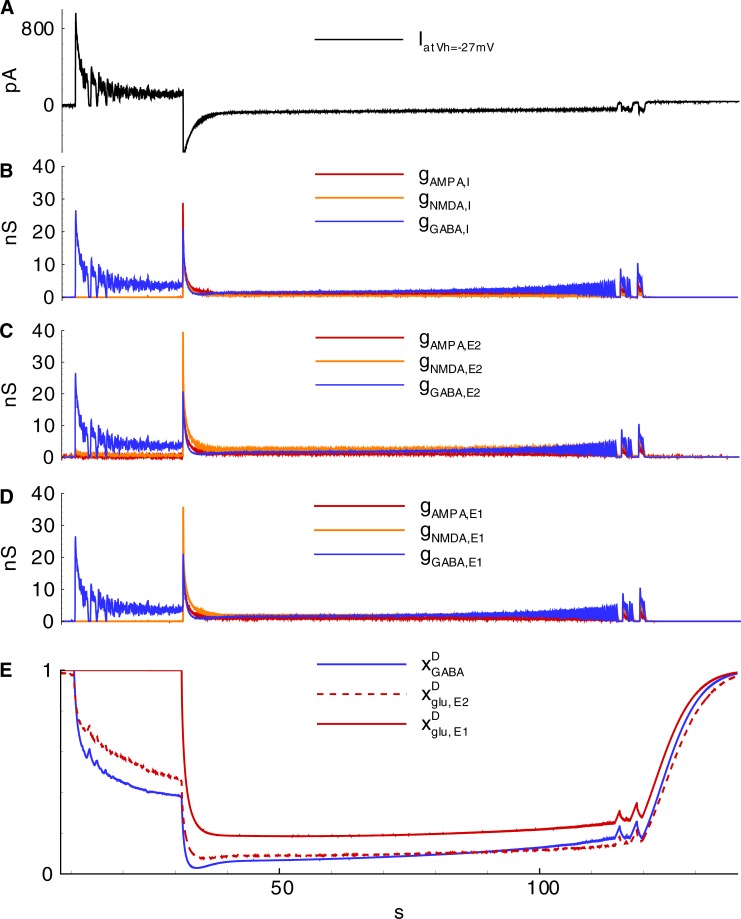
Synaptic conductances and synaptic resource during IID1s and ID are shown in Fig 4 and Fig 9 (Simulation 1). **A**, current at -27 mV in a cell of population *E1*; **B-D**, synaptic conductances in *I-*, *E2-*, and *E1*-neurons, correspondingly; **E**, resources of GABA-synapses and glutamate synapses on *E2-* and *E1*-presynaptic neurons.

**Fig 11:**
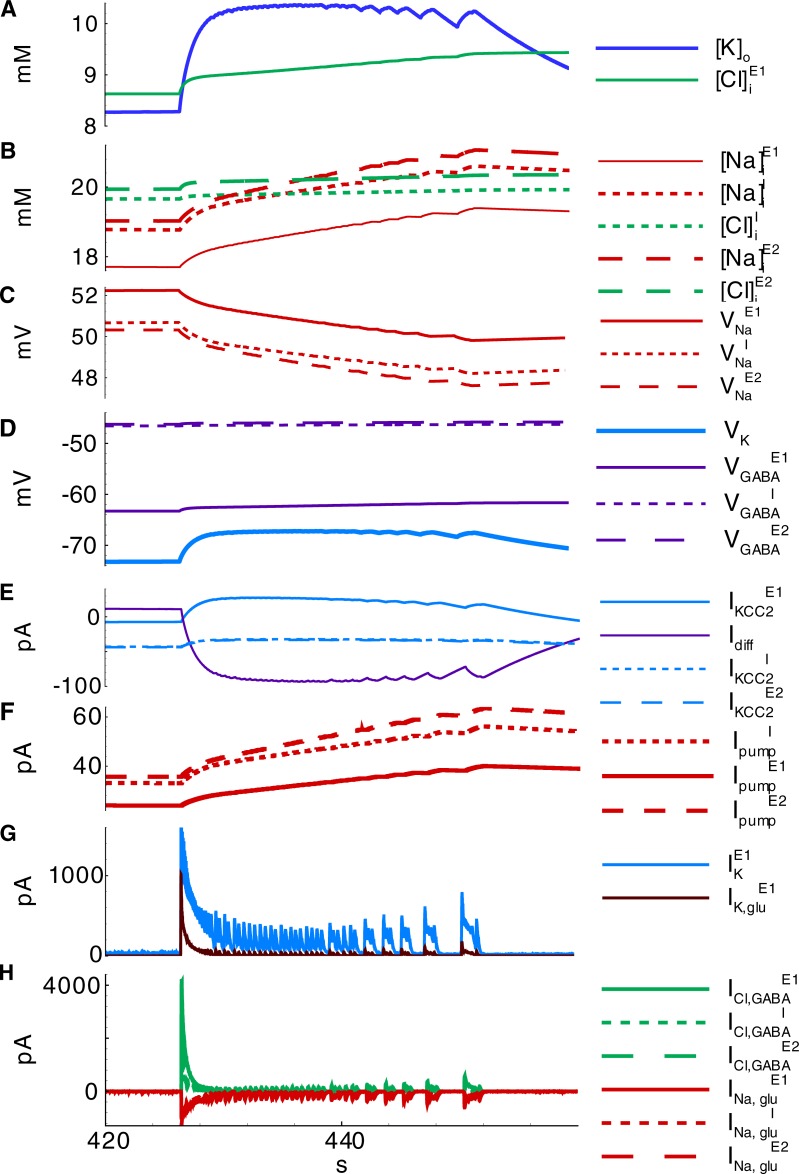
Ionic dynamics during ID shown in Fig 4 and Fig 9 (Simulation 1). **A,B**, ionic concentrations; **C,D**, reversal potentials; **E**, potassium currents due to KCC2 activity and diffusion with bath solution; **F**, Na-K pump activity; **G**, potassium currents through voltage-gated and glutamatergic channels for *E1*-neurons; **H**, chloride current through GABARs and sodium currents through glutamatergic channels.

The accumulation of chloride within the *E1*-population takes place during IID1s (${\left[Cl\right]}_{i}^{E1}$ in Figs 8A and 7F) due to the activation of GABA-A receptors ($I_{Cl,GABA}^{E1}$ in Fig 8E). The reversal potential $V_{GABA}^{E1}$ is getting more depolarized (Fig 9G). A commonly negative driving force $V_{GABA}^{E1}-V_{}^{E1}$ decreases, thus reducing GABAergic inhibition in *E1*-neurons. Moreover, the extracellular potassium concentration increases (Fig 9F) due to the potassium outflow from *E1*-, *E2*-, and *I*-neurons. The enhanced level of extracellular potassium concentration provides a depolarizing effect. An activation of *E1*-neurons by *E2*-neurons through glutamatergic synapses becomes possible. Eventually, an IID1 either switches to an IID2 or an ID (Fig 2B, Fig 9B) with a rapid burst of *E1*-neuron firing (Fig 9E). Such ID generation after a series of IID1s was observed in the experiment and the simulation (Fig 2B and Fig 9B) and as a strong negative component (at the time moment about 31 s) in the current trace registered at the holding voltage -27 mV (Fig 9B). All the synaptic pathways are activated during the generation of IDs (Fig 6A). The *E1*-population fires and thus provides a robust glutamatergic excitation (see peak AMPA and NMDA conductances in Fig 10B–10D). This excitation is observed in the VC mode as a large negative pulse (Fig 4B, Fig 9B). This current pulse is mostly determined by $I_{Na,glu}^{E1}$, $I_{Na,glu}^{I}$, and $I_{Na,glu}^{E2}$ (Fig 11H) and leads to the depolarization of *E1-* and *E2*-neurons by 20 mV (Fig 9D and 9E) as well as the overexcitation of *I*-cells in the form of a depolarization block (Fig 9C). The ID continues with the generation of short bursts (Fig 4D). The short bursts at the end of an ID in Simulation 1 (Fig 4C–4G) were similar to the IID2-like events of Simulation 2 (Fig 5D, 5F, 5H, 5J and 5L).

**Fig 12:**
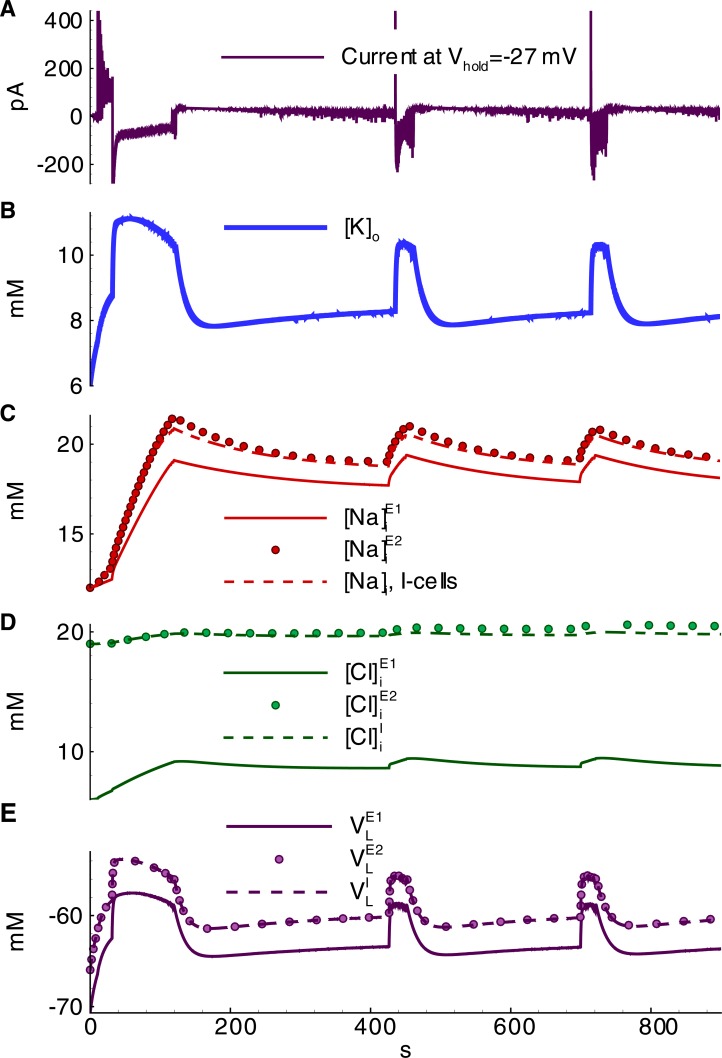
Ionic concentrations and leak reversal potentials during a series of IDs shown in Fig 4 (Simulation 1). **A**, current at -27 mV in a cell of population *E1*; **B**, extracellular potassium concentration; **C,D**, sodium and chloride concentrations in different cells, correspondingly; **E**, leak reversal potentials.

The termination of active discharge generation occurs after the accumulation of intracellular sodium (${\left[Na\right]}_{i}^{E1}$, ${\left[Na\right]}_{i}^{I}$, and ${\left[Na\right]}_{i}^{E2}$ in Figs 12C and 8B), which activates the Na-K-pump ($I_{pump}^{E1}$, $I_{pump}^{I}$, and $I_{pump}^{E2}$ in Fig 11F). In turn, potassium is pumped into the neurons, thus decreasing [*K*]_*o*_ (Fig 11A). Both the decreased [*K*]_*o*_ and the electrotonic effect of the Na-K pump repolarize the neurons. The spike generation decays (Fig 4E–4G) through the transition to IID2 generation. The average firing rate decreases. Consequently, the potassium currents decrease (Fig 11G). The activity of the Na-K pump and the decreased potassium outflux through voltage-gated and synaptic channels accelerate the decrease of [*K*]_*o*_ (Fig 11A, Fig 12B). Consecutive repolarization terminates the ID.

After an ID is terminated, the sodium influx decays such that along with the action of the Na-K pump, it leads to the gradual decrease of the intracellular sodium concentration (Fig 11B, Fig 12C). The activity of the Na-K pump diminishes. [*K*]_*o*_ begins to increase again (Fig 12B). Finally, it results in the generation of another ID (Fig 12). The exhaustion of a synaptic resource is one of the main processes that shape IIDs and IDs. For the regime with IID1s, the GABAergic conductance significantly decays after the first IID1s (Fig 10B, 10C and 10D) due to a reduction in the resource $x_{GABA}^{D}$ (Fig 10E); however, these synapses are still effective up to the moment of the ID onset. At this point, the glutamatergic synapses are full of synaptic resources $x_{glu,E1}^{D}$ and $x_{glu,E2}^{D}$. Hence, the peak NMDA-conductance is high. Quite rapidly, $x_{glu,E1}^{D}$, $x_{glu,E2}^{D}$, and $x_{GABA}^{D}$ reduce. Consequently, GABA-A-, AMPA-, and NMDA-conductances become small. The principle *E1*-cells retain the most abundant resource and are the most effective during the mid-stage of an ID. During the late stage of an ID, the synaptic resources become restored and begin to shape IID2-like events. A few seconds after the end of an ID, the synaptic resources become completely restored (Fig 10E).

Meanwhile, the termination of the electrogenic effect of the Na-K pump after an ID is a slow process (Fig 11F) along with the restoration processes of sodium and potassium concentrations (Fig 11A and 11B, Fig 12B and 12C). These processes determine the intervals between IDs. The cumulative effect of the concentrations is expressed by changes in the leak reversal potential (Fig 12E) that governs the level of the resting membrane potential and consequently the probability of spontaneous cell firing. To a large extent, the level of the leak reversal potential determines the moment of the next ID.

#### Heterogeneity of cell population

**Fig 13:**
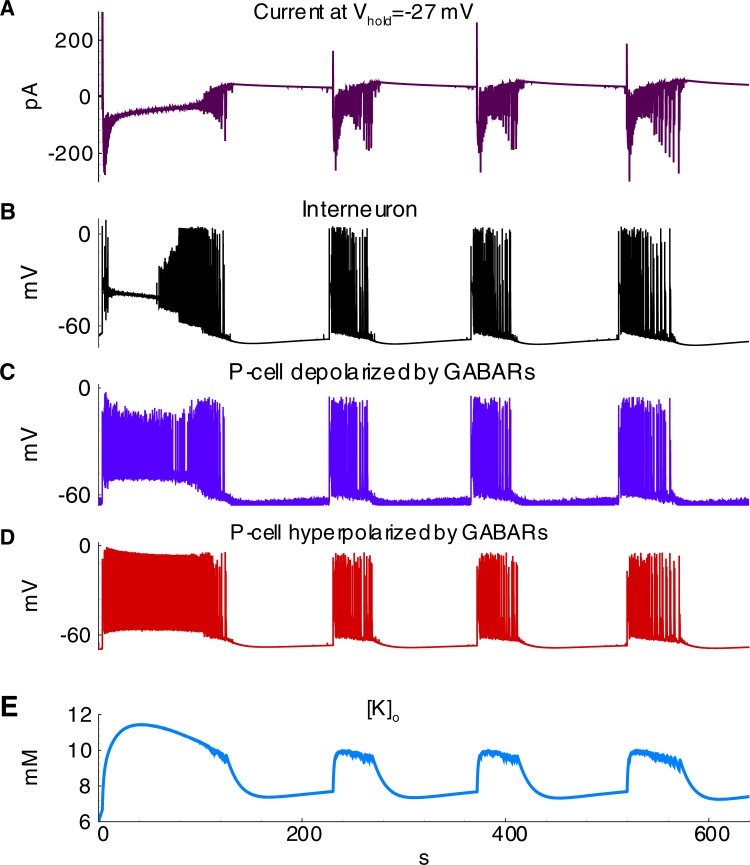
Ictal discharges in the simulation with equal contributions of E1 and E2 cells (Simulation 3). **A**, current recorded in a pyramidal cell (population E1) at the hold voltage -27mV; **B**-**D**, the membrane potential of cells representing interneurons (population *I*), pyramidal cells depolarized by GABARs (population *E2*) and pyramidal cells hyperpolarized by GABARs (population *E1*); **E**, extracellular potassium concentration.

The heterogeneity of the neuronal population is an essential factor in the mechanism of ID generation. Depolarized by GABA receptors, neurons support the generation of IID-like events, whereas the involvement of hyperpolarized neurons by GABA receptors leads to a massive firing. For the proposed model, the heterogeneity was set by splitting the population of excitatory neurons into two subpopulations of *E1* and *E2* cells. The ratio of fractions of *E1* to *E2* neurons within the entire population of pyramidal neurons influences the characteristics of IDs. In a simulation with equal fractions of *E1* and *E2* cells (Simulation 3), the discharges were longer and more frequent than in the case of the basic ratio of 6.5:1 (see *α*_*E*1_/*α*_*E*2_ in Methods), as can be observed from a comparison of Fig 13 with Fig 4. Therefore, a larger number of cells with an impaired level of chloride creates a pro-epileptic effect.

#### Effects of other parameters

**Fig 14:**
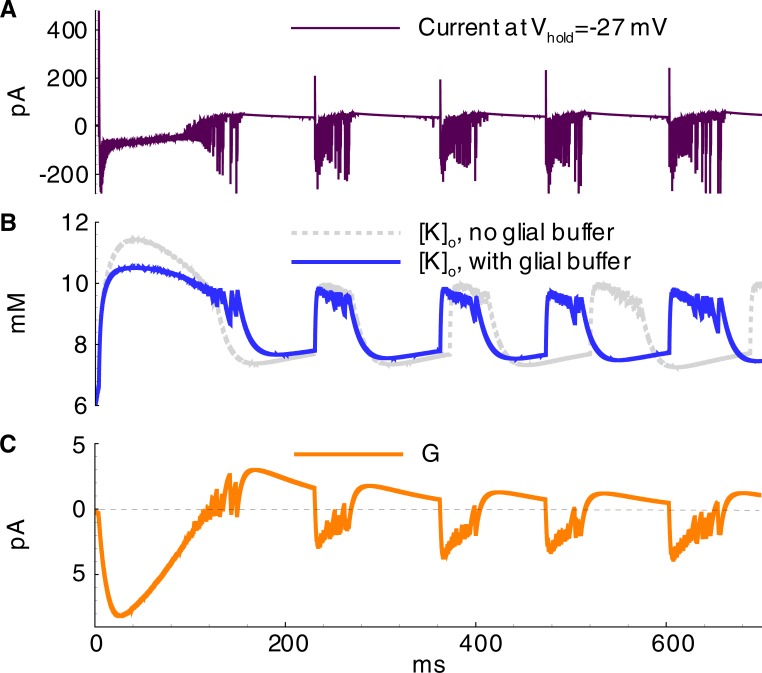
Ictal discharges in the simulation with equal contributions of E1 and E2 cells and active glial buffer (k_1_ = 0.01s^-1^) (Simulation 4). **A**, current recorded in a pyramidal cell (population E1) at the hold voltage -27mV; **B**, extracellular potassium concentration; **C**, the current through glial buffer given by Eqs (16,17).

**Fig 15:**
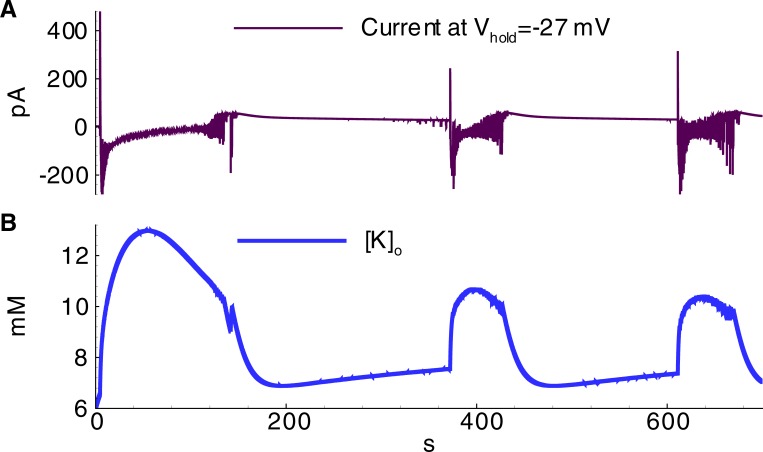
Ictal discharges in the simulation with equal contributions of E1 and E2 cells and weaker diffusion (D = 0.02s^-1^) (Simulation 5). **A**, current recorded in a pyramidal cell (population E1) at the hold voltage -27mV; **B**, extracellular potassium concentration.

The effects of the primary model parameters on the hyperexcitability of neurons and networks have been previously studied [5,6,11,14,45]. The proposed model reproduced some of the effects; however, a systematic parametric analysis is beyond the scope of the present study, though the results of the mentioned studies were applied for setting and tuning the model. For instance, based on research conducted by Cressman et al. [6], a robust glial buffer could eliminate seizures, whereas to the other extreme, an omission of a moderate glial buffer should not qualitatively change the regime of sodium and potassium oscillations during ID generation. Therefore, the glial buffer was not included in the basic set of parameters, but its effect was tested in an auxiliary simulation. In a simulation with the glial buffer, a higher frequency of IDs and a smaller magnitude of [*K*]_*o*_ oscillations were observed (compare Fig 14 to Fig 13). The glial buffer is not strong enough to affect the level of [*K*]_*o*_ during the discharges, when the potassium fluxes through voltage-gated and glutamatergic channels are intense. After an ID the potassium is pumped out the glial cells to extracellular space (Fig 14C), thus elevating the minimum level of [*K*]_*o*_ and shortening the rebound stage of potassium evolution between IDs (Fig 14B). Besides, it was observed that a decrease in diffusion increases the magnitude of potassium fluctuations (compare Fig 15 to Fig 13).

## Discussion

In the present study, based on experimental recordings in combined hippocampal-entorhinal cortex slices in a high-potassium and low-magnesium solution containing 4-AP, a computational model has been developed that incorporates sodium, potassium, and chloride ion concentration dynamics. The findings help elucidate the mechanisms of seizure development and termination. In accordance with previous experimental studies [46–51], a positive feedback loop between the extracellular potassium concentration and neuronal activity results in seizures. High levels of neural activity initiate extracellular potassium and intracellular sodium accumulation, which increases the activity of the Na-K pump and it in turn terminates each ID. This mechanism is consistent with previous modeling works [5,6,12,42]. Significant and novel achievements include: (i) the model reproduces repetitive IDs; (ii) the model reproduces a bursting regime under which the IID-like events are embedded in an ID; (iii) the model reproduces IIDs and IID-like events initiated by interneurons; and (iv) contributions of synaptic channels to the ion concentration dynamics have been visualized.

The IIDs have already been simulated with a CBRD model that does not take into account the ionic dynamics [27]. The simulations reflected the experimentally observed two types of IIDs, pure GABAergic and GABA-glutamatergic. In our preparation [16], the GABAR-mediated IID1 properties resembled those of a previously described events referred to as slow interictal potentials [47] or preictal discharges [52,53]. Hypersynchronous GABAR-mediated potentials have also been recorded in several areas of isolated guinea pig brain preparation during 4-AP application [52,53]. These type events are purely GABAergic [16]; when induced by 4-AP, they are minimally affected by NMDA and non-NMDA glutamatergic receptor antagonists [54].

Regarding the details of the mechanisms of neuronal excitability and ionic transport that were included for consideration, the model is similar to a recently published model [1] in which a single ID was simulated with a model that included a pair of excitatory and inhibitory neurons under the conditions of dynamic ionic concentrations. In comparison with this previous study, some of the details were omitted for the proposed model, such as the multi-compartmentalization of neurons, the explicit calcium dynamics, the dynamics of minor extracellular sodium and chloride, and intracellular potassium ion concentrations. Instead, the model considers quite important factors, such as a large number of neurons and synapses, the heterogeneity of neurons with different ionic transporter concentrations, the NMDA conductance voltage dependence, the short-term synaptic depression, the ion fluxes through the glutamatergic synapses, the bicarbonate flux through GABAergic channels, and NKCC1 contribution. The key advantage of the model is the reproduction of repetitive IDs, which have not been presented in the mentioned paper [1].

Repetitive IDs were simulated in the works by Krishnan et al. [12] and Gonzalez et al. [43,55]. Their network model was based on two-type, two-compartment neurons under the conditions of dynamic ionic concentrations. In comparison with their study, the proposed model neglects the calcium dynamics, the inhomogeneity of the potassium concentration along the neuronal compartments, the dynamics of extracellular sodium and chloride, and intracellular potassium ion concentrations, and the set of voltage-gated channels is different. Instead, the proposed model includes the effects of potassium on chloride concentration dependence by means of KCC2 and NKCC1 cotransporters, the ionic fluxes through the synaptic channels, the heterogeneity of neurons with different ionic transporter concentrations, etc. This approach to neuronal population modeling involves infinite numbers of neurons and synapses, which is an abstraction that avoids artifacts occurring in small networks, such as large synaptic current fluctuations. Comparing the results of the two approaches, Krishnan’s model also reproduces repetitive IDs. These IDs are also Na-K-pump-dependent; however, ID termination is crucially dependent on the depolarization block in pyramidal neurons, which was not the case for the simulations of this study. In experiments [16], the depolarization block that occurs during IDs was usually observed only in interneurons. The most recent study by Gonzalez et al. [55] reveals the role of inhibition of A-type potassium channels by 4-AP and hypothesizes a mechanism of pro-epileptic effect of KCC2 cotransporters. According to this mechanism, chloride accumulates inside neurons and activates KCC2, which results in intensive accumulation of [*K*]_*o*_, thus providing the pro-epileptic effect. Qualitatively our simulations do not contradict to their study, however quantitatively the potassium currents through voltage-gated and synaptic channels dominate over the KCC2-mediated potassium fluxes, and initial accumulation [*K*]_*o*_ that triggers spontaneous discharges is evoked rather by the diffusion with the high-potassium bath solution, according to our experimental model of epilepsy. Though, the contribution of KCC2 cotransporters might be effective on large time scales of inter-discharge intervals, after being triggered by the enhanced level of intracellular chloride. More detailed consideration of the pure 4-AP-triggered epilepsy model is to be a focus of the future studies with the proposed model, which, by the way, explicitly takes into account the A-currents.

Effective factors of oxygen concentration and volume dynamics have been considered using a single neuron model under the conditions of dynamic sodium, potassium, and chloride ionic concentrations [13,15] and with a pair of interacting excitatory and inhibitory neurons under the conditions of dynamic sodium and potassium ionic concentrations [14], though the ionic fluxes through synaptic channels have not been taken into account. The oxygen concentration and volume change effects have been revealed in previous works through a bifurcation analysis. These aspects modulate the contribution of faster processes that more directly affect neurons, such as Na-K pump and ionic concentration dynamics, thus effectively widening the parameter domain corresponding to ID generation. These aspects will be included in future studies. In its present state, the model is one of the most detailed among the models presented in the literature; however, it is still missing some aspects, which represents a compromise as well. The parameters chosen for the model provide oscillations of ion concentrations. The ranges of the parameters that allow for oscillations have been identified in [6]. Due to differences in the basic neuron models and the introduction of additional factors, the parameters set are different from those of the mentioned works to some extent; however, the effects of the major factors are similar.

Overall, the experiments and simulations confirm the mechanism of the initiation of epileptiform activity that was proposed by Huberfeld et al. [17] and Pallud et al. [18]. A high potassium concentration level in the bath solution results in impaired chloride homeostasis due to the activities of potassium-chloride cotransporters [56–58], which leads to the depolarizing effect of GABAergic synapses and thus interneurons and the membrane depolarization in a fraction of neurons. Some depolarized pyramidal cells fire spontaneously. The resulting weak excitation along with GABAergic depolarization leads to excitation and synchronization of the interneurons along with the depolarized pyramidal neuron subpopulation. This synchronization results in IID1s. The cotransporters do not balance chloride influx during an IID1. The duration of such events is sufficient for a millimolar increase in the chloride concentration, whereas transporter-mediated recovery takes minutes [21,38]. Thus, it leads to chloride accumulation and an elevation of the resting membrane potential of pyramidal cells of the hyperpolarized subpopulation. Gradually, a threshold of excitation for this subpopulation decreases. At some point, they start to discharge, beginning as either IID2s or IDs.

Changes in the concentrations of extracellular potassium and intracellular chloride and sodium ions play the most critical roles in seizure induction and maintenance [48]. In accordance with the experimental observations and simulations [5,11,48–50,59], in this model [*K*]_*o*_ had a peak in the middle of an ID, while the intracellular sodium [48] and chloride [22] concentrations still gradually increased until the end of an ID. A high sodium concentration activated the Na-K pump. The fact that the Na-K pump activation is maximal during an ID is indirectly supported with the evidence of a decrease in the oxygen concentration during an ID, which reflected the activity of energy metabolism [60]. The influx of potassium through the Na-K pump overcame the outflux through voltage-gated, leak and synaptic channels and [*K*]_*o*_ began to decrease. The decrease of [*K*]_*o*_ increased the potassium voltage-dependent and leak currents and the termination of the ID. Therefore, the model qualitatively reproduced the ionic dynamics and highlighted the roles of the main factors: the Na-K pump, voltage-gated potassium channels, glutamatergic and GABAergic channels, KCC2 cotransporters, and potassium ion diffusion.

The mechanisms of IID and ID generation in the present model were roughly consistent with those reproduced in the previous simple model, Epileptor-2 [61], where only one population of neurons was explicitly described in terms of the membrane potential along with extracellular potassium, intracellular sodium concentrations, and the synaptic resource. Both the simple and the proposed full model reproduced repeated IDs with each ID representing IID-like events. The IID-like events were large amplitude stochastic oscillations [62]. The IDs were slow oscillations of mainly potassium and sodium ionic concentrations. Contrary to Epileptor-2, the proposed model introduces three populations, includes chloride concentration dynamics, and defines the kinetics more accurately. These details led to the reproduction of the initiation of IDs through chloride impairment due to a high bath concentration of potassium ions and the chloride accumulation following a series of IIDs. In total, this detailed investigation supports the central assumptions of the Epileptor-2 model.

To conclude, this study highlights the importance of neuronal heterogeneity and ionic transport mechanisms in the generation of epileptic discharges.

## Supporting information

S1 Appendix CBRD-approach for populations of pyramidal neurons and interneurons. (PDF)

## References

[1] Gentiletti D, Suffczynski P, Gnatkovsky V, de Curtis M. Changes of Ionic Concentrations During Seizure Transitions—A Modeling Study. Int J Neural Syst. 2017;27(4): 1750004 (16 pages). 10.1142/S0129065717500046 27802792

[2] Kager H, Wadman WJ, Somjen GG. Simulated seizures and spreading depression in a neuron model incorporating interstitial space and ion concentrations. J Neurophysiol. 2000;84(1): 495–512. 10.1152/jn.2000.84.1.495 10899222

[3] Kager H, Wadman WJ, Somjen GG. Conditions for the triggering of spreading depression studied with computer simulations. J Neurophysiol. 2002;88(5): 2700–2712. 10.1152/jn.00237.2002 12424305

[4] Kager H, Wadman WJ, Somjen GG. Seizure-like afterdischarges simulated in a model neuron. J Comput Neurosci. 2007;22(2): 105–128. 10.1007/s10827-006-0001-y 17053996

[5] Cressman JR, Ullah G, Ziburkus J, Schiff SJ, Barreto E. The influence of sodium and potassium dynamics on excitability, seizures, and the stability of persistent states: I. Single neuron dynamics. J Comput Neurosci. 2009;26(2): 159–170. 10.1007/s10827-008-0132-4 19169801 PMC2704057

[6] Ullah G, Cressman JR Jr, Barreto E, Schiff SJ. The influence of sodium and potassium dynamics on excitability, seizures, and the stability of persistent states. II. Network and glial dynamics. J Comput Neurosci. 2009;26(2): 171–183. 10.1007/s10827-008-0130-6 19083088 PMC2951284

[7] Owen JA, Barreto E, Cressman JR. Controlling seizure-like events by perturbing ion concentration dynamics with periodic stimulation. PLoS One. 2013;8(9): e73820 10.1371/journal.pone.0073820 24066075 PMC3774776

[8] Bazhenov M. Potassium Model for Slow (2–3 Hz) In Vivo Neocortical Paroxysmal Oscillations. J Neurophysiology. 2004;92: 1116–1132. 10.1152/jn.00529.2003 PMC2925854 15056684

[9] Bazhenov M, Timofeev I, Steriade M, Sejnowski TJ. Potassium model for slow (2–3Hz) in vivo neocortical paroxysmal oscillations. J Neurophysiol. 2004;92(2): 1116–1132. 10.1152/jn.00529.2003 15056684 PMC2925854

[10] Frohlich F, Bazhenov M, Iragui-Madoz V, Sejnowski TJ. Potassium Dynamics in the Epileptic Cortex: New Insights on an Old Topic. Neuroscientist. 2007;14: 422–433. 10.1177/1073858408317955 PMC2854295 18997121

[11] Krishnan GP, Bazhenov M, Ionic dynamics mediate spontaneous termination of seizures and postictal depression state. J Neurosci. 2011;31(24): 8870–8882. 10.1523/JNEUROSCI.6200-10.2011 21677171 PMC3163257

[12] Krishnan GP, Filatov G, Shilnikov A, Bazhenov M. Electrogenic properties of the Na/K ATPase control transitions between normal and pathological brain states. J Neurophysiol. 2015;113: 3356–3374. 10.1152/jn.00460.2014 25589588 PMC4443608

[13] Wei Y, Ullah G, Schiff SJ. Unification of neuronal spikes, seizures, and spreading depression. J Neurosci. 2014;34(35): 11733–11743. 10.1523/JNEUROSCI.0516-14.2014 25164668 PMC4145176

[14] Wei Y, Ullah G, Ingram J, Schiff SJ. Oxygen and seizure dynamics: II. Computational modeling. J Neurophysiol. 2014;112: 213–223. 10.1152/jn.00541.2013 24671540 PMC4064403

[15] Ullah G, Wei Y, Dahlem MA, Wechselberger M, Schiff SJ. The role of cell volume in the dynamics of seizure, spreading depression, and anoxic depolarization, PLoS Comput Biol. 2015;11(8): e1004414 10.1371/journal.pcbi.1004414 26273829 PMC4537206

[16] Amakhin DV, Ergina JL, Chizhov AV, Zaitsev AV. Synaptic Conductances during Interictal Discharges in Pyramidal Neurons of Rat Entorhinal Cortex. Front Cell Neurosci. 2016;10: 233 10.3389/fncel.2016.00233 27790093 PMC5061778

[17] Huberfeld G, de la Prida LM, Pallud J, Cohen I, Le Van Quyen M, Adam C. et al Glutamatergic pre-ictal discharges emerge at the transition to seizure in human epilepsy. Nature Neuroscience. 2011;14: 627–634. 10.1038/nn.2790 21460834

[18] Pallud J, Le Van Quyen M, Bielle F, Pellegrino C, Varlet P, Labussiere M et al Cortical GABAergic excitation contributes to epileptic activities around human glioma. Science Translational Medicine. 2014;6: 244ra89–244ra89. 10.1126/scitranslmed.3008065 25009229 PMC4409113

[19] Uva L, Breschi GL, Gnatkovsky V, et al Synchronous inhibitory potentials precede seizure-like events in acute models of focal limbic seizures. J Neurosci 2015;35: 3048–3055. 10.1523/JNEUROSCI.3692-14.2015 25698742 PMC6605586

[20] Huberfeld G, Wittner L, Clemenceau S, Baulac M, Kaila K, Miles R, Rivera C. Perturbed chloride homeostasis and GABAergic signaling in human temporal lobe epilepsy. J Neurosci. 2007;27: 9866–9873. 10.1523/JNEUROSCI.2761-07.2007 17855601 PMC6672644

[21] Johansson S, Yelhekar TD, Druzin M. Commentary: Chloride Regulation: A Dynamic Equilibrium Crucial for Synaptic Inhibition. Front Cell Neurosci. 2016;10:182 10.3389/fncel.2016.00182 27487962 PMC4949234

[22] Glykys J, Dzhala V, Egawa K, Balena T, Saponjian Y, Kuchibhotla KV et al Local impermeant anions establish the neuronal chloride concentration. Science. 2014;343(6171): 670–675. 10.1126/science.1245423 24503855 PMC4220679

[23] Eggert J, van Hemmen JL. Modeling neuronal assemblies: Theory and implementation. Neural Computation. 2001;13: 1923–1974. 10.1162/089976601750399254 11516352

[24] Chizhov AV, Graham LJ. Population model of hippocampal pyramidal neurons linking a refractory density approach to conductance-based neurons. Physical Review E. 2007;75: 011924. 10.1103/PhysRevE.75.011924 17358201

[25] Chizhov AV, Graham LJ. Efficient evaluation of neuron populations receiving colored-noise current based on a refractory density method. Physical Review E. 2008;77: 011910. 10.1103/PhysRevE.77.011910 18351879

[26] Chizhov AV, Sanchez-Aguilera A, Rodrigues S, de la Prida LM. Simplest relationship between local field potential and intracellular signals in layered neural tissue. Physical Review E. 2015;92: 062704. 10.1103/PhysRevE.92.062704 26764724

[27] Chizhov AV, Amakhin DV, Zaitsev AV. Computational model of interictal discharges triggered by interneurons. PLoS One. 2017;12(10): e0185752 10.1371/journal.pone.0185752 28977038 PMC5627938

[28] Chizhov AV, Amakhin DV, Zaitsev AV. Spatial propagation of interictal discharges along the cortex. Biochemical and Biophysical Research Communications 2019;508: 1245–1251. 10.1016/j.bbrc.2018.12.070 30563766

[29] Neher E. Correction for liquid junction potentials in patch clamp experiments. Methods Enzymol. 1992;207: 123–131. 1528115 10.1016/0076-6879(92)07008-c

[30] Gerstner W, Kistler WM, Naud R, Paninski L. Neuronal Dynamics From Single Neurons to Networks and Models of Cognition. Cambridge University Press; 2014.

[31] Chizhov AV. Conductance-based refractory density model of primary visual cortex. J Comput Neurosci. 2014;36(2): 297–319. 10.1007/s10827-013-0473-5 23888313

[32] Borg-Graham LJ. Interpretations of Data and Mechanisms for Hippocampal Pyramidal Cell Models In: Cerebral Cortex. Springer Science and Business Media; 1999 pp. 19–138.

[33] Whittington MA, Traub RD, Kopell N, Ermentrout B, Buhl EH. Inhibition-based rhythms: experimental and mathematical observations on network dynamics. Int J Psychophysiology. 2000;38: 315–336. 10.1016/s0167-8760(00)00173-2 11102670

[34] Chizhov AV. Conductance-Based Refractory Density Approach: Comparison with Experimental Data and Generalization to Lognormal Distribution of Input Current. Biol Cybernetics. 2017;111(5–6): 353–364 10.1007/s00422-017-0727-9 28819690

[35] Tsodyks M, Pawelzik K, Markram H. Neural Networks with Dynamic Synapses. Neural Computation. 1998;10(4): 821–835. 9573407 10.1162/089976698300017502

[36] Loebel A, Tsodyks M. Computation by ensemble synchronization in recurrent networks with synaptic depression. J Comp Neuroscience. 2002;13: 111–124. 10.1023/a:1020110223441 12215725

[37] Beaulieu C. Numerical data on neocortical neurons in adult rat, with special reference to the GABA population. Brain Res. 1993;609: 284–292. 10.1016/0006-8993(93)90884-p 8508310

[38] Yelhekar TD, Druzin M, Johansson S. Contribution of Resting Conductance, GABAA-Receptor Mediated Miniature Synaptic Currents and Neurosteroid to Chloride Homeostasis in Central Neurons. eNeuro 2017;4(2): ENEURO.0019-17.2017. 10.1523/ENEURO.0019-17.2017 PMC5362935 28374007

[39] Ostby I, Oyehaug L, Einevoll GT, Nagelhus EA, Plahte E, Zeuthen T, Lloyd CM, Ottersen OP, Omholt SW. Astrocytin mechanisms explaining neural-activity-induced shrinkage of extracneuronal space. PLoS Comput Biol. 2009;5: e1000272 10.1371/journal.pcbi.1000272 19165313 PMC2613522

[40] Rose CR, Konnerth A. NMDA Receptor-Mediated Na1 Signals in Spines and Dendrites. J Neuroscience. 2001;21(12): 4207–4214. 10.1523/JNEUROSCI.21-12-04207.2001 PMC6762772 11404406

[41] Mayer ML, Westbrook GL. Permeation and block of n-methyl-d-aspartic acid receptor channels by divalent cations in mouse cultured central neurones. J Physiology. 1987;394: 501–527. 10.1113/jphysiol.1987.sp016883 PMC1191974 2451020

[42] Barreto E, Cressman JR. Ion concentration dynamics as a mechanism for neuronal bursting. J Biol Phys. 2011;37(3): 361–373. 10.1007/s10867-010-9212-6 22654181 PMC3101327

[43] Gonzalez OC, Shiri Z, Krishnan GP, Myers TL, Williams S, Avoli M, Bazhenov M. Role of KCC2-dependent potassium efflux in 4-Aminopyridine-induced Epileptiform synchronization. Neurobiol Dis. 2018;109(Pt A): 137–147. 10.1016/j.nbd.2017.10.011 29045814 PMC5710807

[44] Jensen MS, Yaari Y. Role of intrinsic burst firing, potassium accumulation, and electrical coupling in the elevated potassium model of hippocampal epilepsy. J Neurophysiol. 1997;77(3): 1224–33. 10.1152/jn.1997.77.3.1224 9084592

[45] Ho ECY, Truccolo W. Interaction between synaptic inhibition and glial-potassium dynamics leads to diverse seizure transition modes in biophysical models of human focal seizures. J Comput Neurosci. 2016;41(2): 225–44. 10.1007/s10827-016-0615-7 27488433 PMC5002283

[46] Fertziger AP, Ranck JB Jr. Potassium accumulation in interstitial space during epileptiform seizures. Exp Neurol. 1970;26(3): 571–85. 5435740 10.1016/0014-4886(70)90150-0

[47] Avoli M, D'Antuono M, Louvel J, Kohling R, Biagini G, Pumain R. et al Network and pharmacological mechanisms leading to epileptiform synchronization in the limbic system in vitro. Prog Neurobiol. 2002;68(3): 167–207. 12450487 10.1016/s0301-0082(02)00077-1

[48] Raimondo JV, Burman RJ, Katz AA, Akerman CJ. Ion dynamics during seizures. Front Cell Neurosci. 2015;9: 419 10.3389/fncel.2015.00419 26539081 PMC4612498

[49] Antonio LL, Anderson ML, Angamo EA, Gabriel S, Klaft ZJ, Liotta A, et al In vitro seizure-like events and changes in ionic concentration. J Neurosci Methods. 2016;260: 33–44. 10.1016/j.jneumeth.2015.08.014 26300181

[50] Avoli M, de Curtis M, Gnatkovsky V, Gotman J, Köhling R, Lévesque M, et al Specific imbalance of excitatory/inhibitory signaling establishes seizure onset pattern in temporal lobe epilepsy. J Neurophysiol. 2016;115(6): 3229–3237. 10.1152/jn.01128.2015 27075542 PMC4946603

[51] de Curtis M, Avoli M. GABAergic networks jump-start focal seizures. Epilepsia. 2016;57(5): 679–687. 10.1111/epi.13370 27061793 PMC4878883

[52] Uva L, Breschi GL, Gnatkovsky V, Taverna S, de Curtis M. Synchronous inhibitory potentials precede seizure-like events in acute models of focal limbic seizures. J Neurosci. 2015;35(7): 3048–55. 10.1523/JNEUROSCI.3692-14.2015 25698742 PMC6605586

[53] Gnatkovsky V, Librizzi L, Trombin F, deCurtis M.Fast activity at seizure onset is mediated by inhibitory circuits in the entorhinal cortex in vitro. Ann.Neurol. 2008;64: 674–686. 10.1002/ana.21519 19107991

[54] Avoli M, deCurtis M. GABAergic synchronization in the limbic system and its role in the generation of epileptiform activity. Prog.Neurobiol. 2011;95: 104–132. 10.1016/j.pneurobio.2011.07.003 21802488 PMC4878907

[55] Gonzalez OC, Krishnan GP, Chauvette S, Timofeev I, Sejnowski T, Bazhenov M. Modeling of Age-Dependent Epileptogenesis by Differential Homeostatic Synaptic Scaling. J Neurosci. 2015;35(39): 13448–62. 10.1523/JNEUROSCI.5038-14.2015 26424890 PMC4588612

[56] Kaila K, Voipio J, Paalasmaa P, Pasternack M, Deisz RA. The role of bicarbonate in GABAA receptor-mediated IPSPs of rat neocortical neurones. J Physiol. 1993;464: 273–289. 8229801 10.1113/jphysiol.1993.sp019634 PMC1175385

[57] Payne JA, Rivera C, Voipio J, Kaila K. Cation-chloride cotransporters in neuronal communication, development and trauma. Trends Neurosci. 2003;26: 199–206. 10.1016/S0166-2236(03)00068-7 12689771

[58] Buchin A, Chizhov A, Huberfeld G, Miles R, Gutkin BS. Reduced Efficacy of the KCC2 Cotransporter Promotes Epileptic Oscillations in a Subiculum Network Model. J Neurosci. 2016;36(46): 11619–11633. 10.1523/JNEUROSCI.4228-15.2016 27852771 PMC6231544

[59] Librizzi L., Losi G., Marcon I., Sessolo M., Scalmani P., Carmignoto G., de Curtis M. Interneuronal network activity at the onset of seizure-like events in entorhinal cortex slices. J Neuroscience. 2017: 3906–3916. 10.1523/JNEUROSCI.3906-16.2017. PMC6596630 28947576

[60] Jirsa VK, Stacey WC, Quilichini PP, Ivanov AI, Bernard C. On the nature of seizure dynamics. Brain. 2014;137(8): 2210–2230. 24919973 10.1093/brain/awu133 PMC4107736

[61] Chizhov AV, Zefirov AV, Amakhin DV, Smirnova EYu, Zaitsev AV. Minimal model of interictal and ictal discharges “Epileptor-2”. PLoS CB. 2018;14(5): e1006186. 10.1371/journal.pcbi.1006186 PMC6005638 29851959

[62] Bashkirtseva I, Fedotov S, Ryashko L, Slepukhina E. Stochastic Bifurcations and Noise-Induced Chaos in 3D Neuron Model. Int J Biffurcation and Chaos 2016;26(12): 1630032–21.

