# Supplementary material for: Mathematical model of Na-K-Cl homeostasis in ictal and interictal discharges
Source: PLoS One. 2019 Mar 15;14(3):e0213904. doi: 10.1371/journal.pone.0213904 (PMC6420042; doi:10.1371/journal.pone.0213904)
Supplement: S1 Appendix — (PDF) [file pone.0213904.s001.pdf]

S1 Appendix

CBRD-approach for populations of pyramidal neurons and interneurons

CBRD-approach for a population of pyramidal neurons

The model of synaptically interacting neuronal populations is based on our previous study [1]. We consider two excitatory and one inhibitory neuronal populations, denoted by indexes *E1*, *E2* and *I*, correspondingly. The types of synapses are denoted by the types of mediator (AMPA, GABA or NMDA), and pre- and postsynaptic neurons.

A mathematical description of each single population is based on the probability density approach [2], namely, the conductance-based refractory density (CBRD) approach [3]. The approach considers a population of an infinite number of Hodgkin-Huxley-like neurons receiving both a common input and an individual for each neuron noise. In any arbitrary case of transient or steady-state stimulation the firing rate of such population can be quite precisely and computationally effectively calculated by solving a system of equations in partial derivatives, 1-d transport equations. The equations govern an evolution of neuronal states distributed in a phase space of the time elapsed since last spikes, t^*. They contain the Hodgkin-Huxley equations for the membrane voltage and gating variables, parameterized by t^*, as well as the equation for the neuronal density in t^*-space, $\rho^E(t,t^*)$, where the index *E* substitutes for *E1* or *E2*. The output characteristic of the population's activity is the firing rate $\nu^E(t)$, which is equal to ρ^E in the state of a spike, $t^*=0$. The equations written below describe an excitatory population of adaptive regular spiking pyramidal cells according to our previous works [3,4].

Basic neurons have 2-compartments with the somatic and dendritic voltages $U^E(t,t^*)$ and $U_d^E(t,t^*)$. In comparison with one-compartment model, the extra parameters is the ratio of dendritic to somatic conductances γ and the dendritic length. The inhibitory synapses are located at soma, contributing into the somatic synaptic current I_{soma}, whereas the excitatory synapses are at dendrites, determining the dendritic synaptic current I_{dendr}. Due to the construction of the 2-compartment model [5], both type synaptic conductances are imposed to be somatic, in spite of the localization, in order to be compared with experimental whole-cell somatic registrations. Approximations of voltage-gated ionic currents are based on the CA1 pyramidal cell model from [6], where instead of full description of calcium dynamics and calcium-dependent potassium currents a cumulative after-spike hyperpolarization (AHP) current, that provides an effect of slow spike timing adaptation [7]. Parameterized by t^*, the governing equations are as follows:

$$\frac{\partial \rho^E}{\partial t}+\frac{\partial \rho^E}{\partial t^*}=-\rho^E H(U^E,g_{tot}^E), \tag{A1}$$

$$C\left(\frac{\partial U^E}{\partial t}+\frac{\partial U^E}{\partial t^*}\right)=-g_L(U^E-V_L)+\frac{2\gamma}{l}g_L(U_d^E-U^E) \\ -I_{DR}-I_A-I_M-I_{AHP}+I_{soma}+I_{noise} \tag{A2}$$

PDF Created with deskPDF PDF Writer - Trial :: http://www.docudesk.com

$$C\left(\frac{\partial U_d^E}{\partial t}+\frac{\partial U_d^E}{\partial t^*}\right)=-g_L(U_d^E-V_{rest})-\frac{2}{l}g_L(U_d^E-U^E)+\frac{I_{dendr}}{\gamma}, \tag{A3}$$

where $g_{tot}^E(t,t^*)$ is the total conductance including the leak, voltage-gated and synaptic conductance $g_{syn}^E(t,U^E)=g_{AMPA,E1,E}(t)+g_{AMPA,E2,E}(t)+g_{NMDA,E1,E}(t,U^E)+g_{NMDA,E2,E}(t,U^E)+g_{GABA,I,E}(t)$ l is the square ratio of the dendritic length to the characteristic length. The somatic and dendritic synaptic currents I_{soma} and I_{dendr} are calculated as

$$I_{soma}=g_{GABA,I,E}(t)(V_{GABA}-U^E)$$

$$I_{dendr}=\left(\frac{l\,\tau_m^0}{2}\frac{d}{dt}+1+\frac{l}{2}\right)\left(\begin{array}{l}(g_{AMPA,E1,E}(t)+g_{AMPA,E2,E}(t))(V_{AMPA}-U^E)\\+(g_{NMDA,E1,E}(t)+g_{NMDA,E2,E}(t))(V_{NMDA}-U^E)\end{array}\right),$$

where the differential operator represents the solution of the reverse problem of dendritic current estimation from somatically registered-like conductances [5]. The synaptic conductance kinetics is estimated from somatic responses to stimulation of presynaptic neuronal population, thus it implicitly accounts not only the kinetics of synaptic channels but also the dendritic and axonal propagation delays. For the dendritic compartment, the differential operator sharpens the transient effect of the channels, thus providing better agreement between somatic postsynaptic currents and potentials. This sharpening affects only glutamatergic channels located on the dendritic compartment.

Hazard function

The source term in the eq.(A1) is the hazard function H which is defined as the probability for a single neuron to generate a spike, if known actual neuron state variables. The approximation of the hazard function H has been obtained for the case of white noise [3] and color noise [4] as a function of $U(t)$, $g_{tot}^E(t,t^*)$, and parameters of the noise amplitude in the resting state σ_V^0, the spike threshold voltage V_{th}, and the ratio of the membrane time constant $\tau_m=C/g_{tot}$ to the noise time constant τ_{Noise}, $k=\tau_m/\tau_{Noise}$:

$$H(U)=A+B, \tag{A4}$$

$$A=\frac{1}{\tau_m}e^{0.0061-1.12\,T-0.257\,T^2-0.072\,T^3-0.0117\,T^4}(1-(1+k)^{-0.71+0.0825(T+3)}),$$

$$B=\sqrt{2}\left[-\frac{dT}{dt}\right]_+\sqrt{\frac{2}{\pi}}\frac{\exp(-T^2)}{1+erf(T)},\quad T=\frac{V_{th}-U}{\sqrt{2}\,\sigma_V}\sqrt{\frac{g_{tot}^E}{g_L}},$$

where T is the membrane potential relative to the threshold, scaled by the noise amplitude σ_V which increases with the synaptic conductance as $\sigma_V=\sigma_V^0\sqrt{1+g_{syn}/g_L}$. The term A is the hazard for a neuron to cross the threshold because of noise, derived analytically [3] and approximated by exponential and polynomial for convenience; B is the hazard for a neuron to fire because of depolarization due to deterministic drive, i.e. the hazard due to drift in the voltage phase space. Note that the H-function is independent of the basic neuron model and does not contain any free parameters or functions for fitting to any particular case. Thus, H-function is the same for excitatory and inhibitory populations.

Voltage-dependent channels

PDF Created with deskPDF PDF Writer - Trial :: http://www.docudesk.com

The set of ionic currents includes the voltage-dependent potassium currents I_{DR} and I_A responsible for spike repolarization, the slow potassium current I_M that contributes to spike frequency adaptation and the potassium current I_{AHP}, implicitly dependent on calcium dynamics and contributing to spike frequency adaptation. Approximating formulas for the currents I_{Na}, I_{DR}, I_A and I_M are taken from [6]; the approximation for I_{AHP} is given in [7].

The voltage-dependent potassium current I_{DR}:

$$I_{DR}(U^E,t,t^*) = \bar{g}_{DR}\, x(t)\, y(t)\,(U^E(t) - V_K), \quad \text{(A5)}$$

$$\frac{\partial x}{\partial t} + \frac{\partial x}{\partial t^*} = \frac{x_\infty(U^E) - x}{\tau_x(U^E)}, \quad \text{(A6)}$$

$$\frac{\partial y}{\partial t} + \frac{\partial y}{\partial t^*} = \frac{y_\infty(U^E) - y}{\tau_y(U^E)} \quad \text{(A7)}$$

$$\tau_x = 1/(a+b) + 0.8\ ms;$$
$$x_\infty = a/(a+b),$$
$$a = 0.17\exp((U^E + 5)\cdot 0.090)\ ms^{-1},$$
$$b = 0.17\exp(-(U^E + 5)\cdot 0.022)\ ms^{-1},$$
$$\tau_y = 300\ ms,$$
$$y_\infty = 1/(1 + \exp((U^E + 68)\cdot 0.038));$$

The voltage-dependent potassium current I_A:

$$I_A(U^E,t,t^*) = \bar{g}_A\, x^4(t)\, y^3(t)\,(U^E(t) - V_K), \quad \text{(A8)}$$

$$\frac{\partial x}{\partial t} + \frac{\partial x}{\partial t^*} = \frac{x_\infty(U^E) - x}{\tau_x(U^E)}, \quad \text{(A9)}$$

$$\frac{\partial y}{\partial t} + \frac{\partial y}{\partial t^*} = \frac{y_\infty(U^E) - y}{\tau_y(U^E)} \quad \text{(A10)}$$

$$\tau_x = 1/(a_x + b_x) + 1\ ms;$$
$$x_\infty = a_x/(a_x + b_x),$$
$$a_x = 0.08\exp((U^E + 41)\cdot 0.089)\ ms^{-1},$$
$$b_x = 0.08\exp(-(U^E + 41)\cdot 0.016)\ ms^{-1},$$
$$\tau_y = 1/(a_y + b_y) + 2\ ms;$$
$$y_\infty = a_y/(a_y + b_y),$$
$$a_y = 0.04\cdot\exp(-(U^E + 49)\cdot 0.11)\ ms^{-1},$$
$$b_y = 0.04\ ms^{-1};$$

The voltage-dependent potassium current I_M:

$$I_M(U^E,t,t^*) = \bar{g}_M\, x^2(t)\, y(t)\,(U^E(t) - V_K), \quad \text{(A11)}$$

$$\frac{\partial x}{\partial t} + \frac{\partial x}{\partial t^*} = \frac{x_\infty(U^E) - x}{\tau_x(U^E)}, \quad \text{(A12)}$$

$$\frac{\partial y}{\partial t} + \frac{\partial y}{\partial t^*} = \frac{y_\infty(U^E) - y}{\tau_y(U^E)} \quad \text{(A13)}$$

PDF Created with deskPDF PDF Writer - Trial :: http://www.docudesk.com

$$\tau_x = 1/(a+b) + 8\ ms,$$
$$x_\infty = a/(a+b),$$
$$a = 0.003\exp((U^E + 45)\cdot 0.135)\ ms^{-1},$$
$$b = 0.003\exp(-(U^E + 45)\cdot 0.090)\ ms^{-1},$$
$$\tau_y = 1000\ ms,$$
$$y_\infty = 1/(1+\exp((U^E + 40)/5));$$

The adaptation current I_{AHP}:

$$I_{AHP}(U^E, t, t^*) = \bar{g}_{AHP}\ x(t)\,y(t)\,(U^E(t) - V_K), \quad \text{(A14)}$$
$$\frac{\partial x}{\partial t} + \frac{\partial x}{\partial t^*} = \frac{x_\infty(U^E) - x}{\tau_w(U^E)}, \quad \text{(A15)}$$
$$\frac{\partial y}{\partial t} + \frac{\partial y}{\partial t^*} = \frac{y_\infty(U^E) - y}{\tau_y(U^E)} \quad \text{(A16)}$$
$$\tau_w = 2000/(3.3\exp((U^E + 35)/20) + \exp(-(U^E + 35)/20))\ ms,$$
$$x_\infty = 1/(1+\exp(-(U^E + 35)/4)),$$
$$\tau_y = 1000\ ms,$$
$$y_\infty = 1/(1+\exp((U^E + 40)/5));$$

Boundary conditions.

According to the conservation of the number of neurons in a population, the firing rate is calculated as a sink of neurons from their state t^* due to spiking, $\rho^E(t,t^*)\,H(U^E(t,t^*)$, integrated over the whole phase space, i.e.

$$\nu^E(t) \equiv \rho^E(t,0) = \int_{+0}^{\infty} \rho^E(t,t^*)\,H(U^E(t,t^*))dt^*. \quad \text{(A17)}$$

It is the boundary condition for eq.(A1).

The spike duration is taken into account by introducing the time interval $0 < t^* < \Delta t_{AP}$ during which the voltage and the gating variables are fixed to their reset values. It defines the boundary conditions for eqs.(A2-A14) at $t^* = \Delta t_{AP}$ which are as follows:

$$U^E(t, \Delta t_{AP}) = V_{reset}, \quad \text{(A18)}$$
$$U_d^E(t, \Delta t_{AP}) = V_{rest}; \quad \text{(A19)}$$
$$I_{DR}:\ x(t,\Delta t_{AP}) = 0.262,\ \ y(t,\Delta t_{AP}) = 0.473; \quad \text{(A20)}$$
$$I_A:\ x(t,\Delta t_{AP}) = 0.743,\ \ y(t,\Delta t_{AP}) = 0.691. \quad \text{(A21)}$$

The reset values for the fast gating variables in eqs.(A18, A19) were obtained with the basic single neuron model. With a rather arbitrary input providing a spike, these values were measured at the moment of a voltage maximum at the spike. The reset level for each slow conductance in the CBRD model was calculated as a sum of its value at a peak of spike-release distribution in the t^*-space and an increment at spike:

$$I_M:\ x(t,\Delta t_{AP}) = x(t,t^{*p}) + 0.175\,(1 - x(t,t^{*p})),\quad y(t,\Delta t_{AP}) = y(t,t^{*p}) - 0.003\,y(t,t^{*p}); \quad \text{(A22)}$$
$$I_{AHP}:\ x(t,\Delta t_{AP}) = x(t,t^{*p}) + 0.018\,(1 - x(t,t^{*p})),\quad y(t,\Delta t_{AP}) = y(t,t^{*p}) - 0.003\,y(t,t^{*p}); \quad \text{(A23)}$$

where t^{*p} is such that

PDF Created with deskPDF PDF Writer - Trial :: http://www.docudesk.com

$$\rho(t,t^{*p})\,H(t,t^{*p}) = \max_{0<t^*<+\infty} \rho(t,t^*)\,H(t,t^*).$$

The increment values for the slow gating variables in eqs.(A22-A23) were also measured at a single spike of the single neuron model.

Parameters

$$\bar{g}_{DR} = 0.76\,\mu S/cm^2,\ \bar{g}_A = 4.36\,\mu S/cm^2,$$
$$\bar{g}_M = 0.76\,\mu S/cm^2,\ \bar{g}_{AHP} = 0.6\,\mu S/cm^2,$$
$$\tau_m^0 = C/g_{tot}^0 = 14.4\ ms,\ (g_L = 0.048\,\mu S/cm^2),$$
$$V_{th}(t^*) = (-40 + 50\exp(-t^*/10\ ms)\ mV,$$
$$V_{reset} = -40\ mV,\ \Delta t_{AP} = 1.5\ ms,$$
$$\gamma = 2.85,\ \ C = 0.7\,\mu F/cm^2,\ \ \sigma_V = 2(1 + g_{syn}/g_{tot}^0)\ mV,$$
$$S = 10^{-4}\ cm^2$$
$$g_L = g_{KL} + g_{ClL} + g_{NaL}\,,\ V_L = \left(V_K\, g_{KL} + V_{Cl}\, g_{ClL} + V_{Na}\, g_{NaL}\right)/\left(g_{KL} + g_{ClL} + g_{NaL}\right).$$

Here g_{tot}^0 is the total somatic conductance at rest, and g_{syn} is the total synaptic conductance; S is the membrane area. The dependence of $V_{th}(t^*)$ is taken from a full single neuron model [3], allowing to take into account the effect of sodium channel inactivation on the threshold dynamics [8]. σ_V is the noise amplitude meaning the dispersion of individual neuron's voltage fluctuations in a stationary state. Its scaling with g_{syn} approximately reflects the fact of the synaptic noise increase with the increase of mean synaptic drive [9]. Stochastic input to *E2*-neurons I_{noise} was modeled as Ornstein-Uhlenbeck process with the time correlation 10 ms and the dispersion 20 pA for the ID-regime simulation and 40 pA for the IID-regime simulation.

The equations for the input synaptic conductances $g_{GABA,E}(t)$, $g_{AMPA,E}(t)$ and $g_{NMDA,E}(t)$ are given below, as well as the values of the reversal potentials.

When calculating the dynamics of a neural population, the integration of eqs.(A2,A3,A5-A16) determines the evolution of the distribution of voltage U^E across t^*. Then, the effect of crossing the threshold and the diffusion due to noise are taken into account by H-function, eq.(A4), substituted into the equation for neuronal density, eq. (A1). The integral eq.(A17) results in the output firing rate $\nu^E(t)$.

CBRD-approach for a population of interneurons

The model for the fast spiking single-compartment interneurons is the reduction of the model of the adaptive regular spiking two-compartment neurons:

$$\frac{\partial\rho^I}{\partial t} + \frac{\partial\rho^I}{\partial t^*} = -\rho^I H(U^I, g_{tot}^I), \tag{A24}$$

$$C\left(\frac{\partial U^I}{\partial t} + \frac{\partial U^I}{\partial t^*}\right) = -g_L(U^I - V_L) - I_{DR} - I_A \tag{A25}$$
$$+\left(g_{AMPA,E1,I}(t) + g_{AMPA,E2,I}(t) + g_{NMDA,E1,I}(t,U^I) + g_{NMDA,E2,I}(t,U^I)\right)(V_{AMPA} - U^I)$$
$$+\, g_{GABA,I}(t)(V_{GABA} - U^I)$$

PDF Created with deskPDF PDF Writer - Trial :: http://www.docudesk.com

The voltage-dependent currents I_{DR} and I_A were approximated by eqs.(A5-A10) with U^I instead of U^E. Boundary conditions are given by

$$\nu^I(t) \equiv \rho^I(t,0) = \int_{+0}^{\infty} \rho^I(t,t^*) H(U^I(t,t^*)) dt^*, \quad \text{(A28)}$$

$$U^I(t,\Delta t_{AP}) = V_{reset} \quad \text{(A29)}$$

and eqs.(A20,A21). Parameters are the same as for excitatory cells, except of larger $\sigma_V = 3(1+g_{syn}/g^0_{tot})\ mV$.

Lognormal distribution of synaptic weights within each population

The CBRD-approach is generalized to the case of lognormal distribution of synaptic weights within each j-population [10]. In this case, instead of equal total synaptic current, neurons receive lognormally distributed current. For the current scaled by its mean across the distribution, x, the distribution is

$$\psi(x) = \frac{\exp(-(\ln x)^2/(2\sigma_{LN}^2))}{\sqrt{2\pi}\,\sigma_{LN}\,x} \quad \text{(A30)}$$

The membrane potential of neurons parameterized with x, U_x^j, can be found as

$$U_x^j(t,t^*) = (U^j(t,t^*) - U_{free}^j(t^*))\,x + U_{free}^j(t^*), \quad \text{(A31)}$$

where $U_{free}^j(t^*)$ is the unperturbed potential defined for zero synaptic input.

The density of neurons parameterized by x and distributed in the phase space t^* is denoted as $\rho_x^j(t,t^*)$. Calculation of $\rho_x^j(t,t^*)$ requires solving of a continuum of eqs.(1) (or eq.(22)) for ρ_x^j instead of ρ^j with $H(U_x^j, dU_x^j/dt)$. The output firing rate is defined as

$$\nu^j(t) = \int_0^{\infty} \rho_x^j(t,0)\,\psi(x)\,dx \quad \text{(A32)}$$

In numerical simulations, we set the parameter of the lognormal distribution $\sigma_{LN} = 0.75$ and discretized the x-space by 15 intervals.

Mean somatic and dendritic membrane potentials are calculated as follows:

$$\overline{U}(t) = \int_0^{\infty} U(t,t^*) \int_0^{\infty} \rho_x(t,t^*)\,\psi(x)\,dx\,dt^* \quad \text{(A33)}$$

and

$$\overline{U_d}(t) = \int_0^{\infty} U_d(t,t^*) \int_0^{\infty} \rho_x(t,t^*)\,\psi(x)\,dx\,dt^*\ . \quad \text{(A34)}$$

Connections

The synaptic conductances are described with the second-order differential equations [11] with introduced synaptic plasticity factors $x^D_{glu,E1}(t)$, $x^D_{glu,E2}(t)$ and $x^D_{GABA}(t)$, i.e. as follows

$$g_{AMPA,i,j}(t) = \overline{g}_{AMPA,i,j}\ m_{AMPA,i,j}(t)\ x^D_{glu,i}(t), \quad \text{(A35)}$$

$$g_{NMDA,i,j}(t,U^j) = \overline{g}_{NMDA,i,j}\ f_{NMDA}(U^j(t))\,m_{NMDA,i,j}(t)\ x^D_{glu,i}(t), \quad \text{(A36)}$$

$$f_{NMDA}(V) = 1/(1+Mg/3.57\exp(-0.062\,V)),$$

PDF Created with deskPDF PDF Writer - Trial :: http://www.docudesk.com

$$g_{GABA,j}(t) = \bar{g}_{GABA,j}\, m_{GABA,j}(t) x^D_{GABA}(t), \quad \text{(A37)}$$
$$for \quad i = E1\, and\ E2; \;\; j = E1, E2\ and\ I$$

Mg is the magnesium (Mg^{2+}) concentration in mM; $m_{s,j}(t)$ is the non-dimensional synaptic conductance which is approximated by the second order ordinary differential equation:

$$\left(\tau_r^{s,j}\tau_d^{s,j}\frac{d^2}{dt^2} + (\tau_r^{s,j} + \tau_d^{s,j})\frac{d}{dt} + 1\right) m_{s,j}(t) = \tau^{s,j}\left(1 - m_{s,j}(t)\right)\varphi_i(t), \quad \text{(A38)}$$

$$\tau^{s,j} = \left(\tau_r^{s,j} - \tau_d^{s,j}\right) \Big/ \left((\tau_d^{s,j}/\tau_r^{s,j})^{\tau_d^{s,j}/(\tau_r^{s,j} - \tau_d^{s,j})} - (\tau_d^{s,j}/\tau_r^{s,j})^{\tau_r^{s,j}/(\tau_r^{s,j} - \tau_d^{s,j})} \right), \quad \text{(A39)}$$
$$if \;\; \tau_r^{s,j} \neq \tau_d^{s,j},$$
$$\tau_r^{s,j} e, \;\; otherwise.$$

Here φ_i is the presynaptic firing rate. In neglect of spatial propagation and temporal delays the presynaptic firing rate is equivalent to the somatic firing rate, i.e. $\varphi_i \equiv \nu_i$. The index s is the synapse type, $s = AMPA$, $GABA$ or $NMDA$; the index $i = E$ for $s = AMPA$ or $NMDA$ and $i = I$ for $s = GABA$; w_{glu} and w_{GABA} are the synaptic weights that change because of short-term plasticity; $\bar{g}_{s,j}$ is the maximum conductance, $\tau_r^{s,j}$ and $\tau_d^{s,j}$ are the rise and decay time constants. We imply that the synaptic time constants are estimated from the somatic responses to the stimulation of a presynaptic neuronal population, thus these time constants characterize not only synaptic channel kinetics but the dendritic and axonal propagation delays as well. The time scale $\tau^{s,j}$ is chosen in the form of eq.(A37) in order to provide independence of the maximum of $g_{s,j}(t)$ on $\tau_r^{s,j}$ and $\tau_d^{s,j}$, when $g_{s,j}(t)$ is evoked by a short pulse of $\varphi_j(t)$.

The parameter values were as follows: $\bar{g}_{AMPA,E1,E1} = \bar{g}_{NMDA,E1,E1} = \bar{g}_{AMPA,E1,E2} = \bar{g}_{NMDA,E1,E2} = \bar{g}_{AMPA,E1,I} = \bar{g}_{NMDA,E1,I} = 0.6$ mS/cm^2, $\bar{g}_{AMPA,E2,E1} = \bar{g}_{NMDA,E2,E1} = \bar{g}_{AMPA,E2,E2} = \bar{g}_{NMDA,E2,E2} = \bar{g}_{AMPA,E2,I} = \bar{g}_{NMDA,E2,I} = 0.1$ mS/cm^2, $\bar{g}_{GABA,E1} = \bar{g}_{GABA,E2} = \bar{g}_{GABA,I} = 1$ mS/cm^2, $V_{AMPA} = V_{NMDA} = 0$, $Mg = 0.25$ mM, $\tau_r^{AMPA,E} = \tau_r^{AMPA,I} = 1.7$ ms, $\tau_d^{AMPA,E} = \tau_d^{AMPA,I} = 8.3$ ms, $\tau_r^{NMDA,E} = \tau_r^{NMDA,I} = 6.7$ ms, $\tau_d^{NMDA,E} = \tau_d^{NMDA,I} = 100$ ms, $\tau_r^{GABA,E} = \tau_r^{GABA,I} = 0.5$ ms, $\tau_d^{GABA,E} = \tau_d^{GABA,I} = 20$ ms.

Synaptic plasticity

The synaptic depression was modeled with the Tsodyks-Markram model [12,13]:

$$\frac{dx^D_{glu,E1}}{dt} = \frac{(1 - x^D_{glu,E1})}{\tau_{glu}} - U_{glu}\, x^D_{glu,E1}\, \varphi_{E1}(t), \quad \text{(A40)}$$

$$\frac{dx^D_{glu,E2}}{dt} = \frac{(1 - x^D_{glu,E2})}{\tau_{glu}} - U_{glu}\, x^D_{glu,E2}\, \varphi_{E2}(t), \quad \text{(A41)}$$

$$\frac{dx^D_{GABA}}{dt} = \frac{(1 - x^D_{GABA})}{\tau_{GABA}} - U_{GABA}\, x^D_{GABA}\, \varphi_I(t), \quad \text{(A42)}$$

with $\tau_{glu} = \tau_{GABA} = 2000$ ms and $U_{glu} = U_{GABA} = 0.04$.

Representative neurons

PDF Created with deskPDF PDF Writer - Trial :: http://www.docudesk.com

Representative neurons of each of the populations were modeled by the basic single neuron model with the same synaptic inputs as for the populations. The model is described by the equations for the membrane voltage, eq. (A2,A3,A5-A16) for *E1* and *E2*-populations, and eq.(A25,A5-A10) for *I*-population, where the sum of partial derivatives were substituted by the total derivative in time *t,* and the sodium current was explicitly present in the right-hand part of eq.(A2) and eq.(A25). The sodium current dependent on voltage *V* was approximated by the 4-state Markov model [6]:

$$I_{Na}(t) = \bar{g}_{Na}\, x_1(t)(V(t) - V_{Na}), \qquad \text{(A43)}$$

$$x_1 + x_2 + x_3 + x_4 = 1,$$

$$\frac{dx_i}{dt} = \sum_{j=0,\, j\neq i}^{4} A_{j,i} x_j - x_i \sum_{j=0,\, j\neq i}^{4} A_{i,j}, \quad i = 1,2,3$$

$$A_{1,2} = 3\, ms^{-1},\ A_{1,3} = f_1^{1,3}(V),\ A_{1,4} = f_1^{1,4}(V),$$

$$A_{2,1} = 0,\ A_{2,3} = f_2^{2,3}(V),\ A_{2,4} = 0,$$

$$A_{3,1} = f_1^{3,1}(V),\ A_{3,2} = 0,\ A_{3,4} = f_2^{3,4}(V),$$

$$A_{4,1} = f_1^{4,1}(V),\ A_{4,2} = 0,\ A_{4,3} = 0$$

$$f_1^{i,j}(V) = \{\tau_{min}^{i,j} + 1/\exp(V - V_{1/2}^{i,j} k^{i,j})\}^{-1},$$

$$f_2^{i,j}(V) = \{\tau_{min}^{i,j} + [(\tau_{max}^{i,j} - \tau_{min}^{i,j})^{-1} + \exp(V - V_{1/2}^{i,j} k^{i,j})]^{-1}\}^{-1},$$

$$\tau_{min}^{1,3} = 1/3\, ms,\ V_{1/2}^{1,3} = -51\, mV,\ k^{1,3} = -2\, mV,$$

$$\tau_{min}^{1,4} = 1/3\, ms,\ V_{1/2}^{1,4} = -57\, mV,\ k^{1,4} = -2\, mV,$$

$$\tau_{min}^{2,3} = 1\, ms,\ V_{1/2}^{2,3} = -53\, mV,\ k^{2,3} = -1\, mV,\ \tau_{max}^{2,3} = 100\, ms,$$

$$\tau_{min}^{3,1} = 1/3\, ms,\ V_{1/2}^{3,1} = -42\, mV,\ k^{3,1} = 1\, mV,$$

$$\tau_{min}^{3,4} = 1\, ms,\ V_{1/2}^{3,4} = -60\, mV,\ k^{3,4} = -1\, mV,\ \tau_{max}^{3,4} = 100\, ms,$$

$$\tau_{min}^{4,1} = 1/3\, ms,\ V_{1/2}^{4,1} = -51\, mV,\ k^{4,1} = 1\, mV.$$

LFP model

Two compartment model of principal neurons (*E1*-population) allows calculation of the local field potential (LFP) originating from dipole-like configuration of membrane currents [5]. The LFP signal measured at the somatic depth, $LFP(t)$, is determined by the somatic and dendritic voltages $U^{E1}(t)$, $U_d^{E1}(t)$ and the firing rate $\nu^{E1}(t)$ as follows:

$$LFP(t) = k_1\left(U_d^{E1}(t) - U^{E1}(t)\right) - k_2\, \nu^{E1}(t), \qquad \text{(A44)}$$

where $k_1 = p/(\sigma\, r_i)$, $k_2 = p\, L\, I_{Na}^{\max}\, \tau_{AP}/2\sigma$ with the density per area of homogeneously distributed neurons p, the specific intracellular resistivity r_i, the conductivity of extracellular medium σ, the maximum sodium current at a spike $I_{Na}^{\max}$, the sipke duration τ_{AP}, and the dendritic length L. These coefficients were estimated as $k_1 = 0.1$, $k_2 = 0.03\, mV \cdot s$.

PDF Created with deskPDF PDF Writer - Trial :: http://www.docudesk.com

References

1. Chizhov AV, Amakhin DV, Zaitsev AV. Computational model of interictal discharges triggered by interneurons. PLoS One. 2017;12(10): e0185752.
2. Gerstner W, Kistler WM, Naud R, Paninski L. Neuronal Dynamics. From Single Neurons to Networks and Models of Cognition. Cambridge University Press; 2014.
3. Chizhov AV, Graham LJ. Population model of hippocampal pyramidal neurons linking a refractory density approach to conductance-based neurons. Physical Review E. 2007;75: 011924.
4. Chizhov AV, Graham LJ. Efficient evaluation of neuron populations receiving colored-noise current based on a refractory density method. Physical Review E. 2008;77: 011910.
5. Chizhov AV, Sanchez-Aguilera A, Rodrigues S, de la Prida LM. Simplest relationship between local field potential and intracellular signals in layered neural tissue. Physical Review E. 2015;92: 062704.
6. Borg-Graham LJ. Interpretations of Data and Mechanisms for Hippocampal Pyramidal Cell Models. In: Cerebral Cortex. Springer Science and Business Media; 1999. pp. 19-138.
7. Whittington MA, Traub RD, Kopell N, Ermentrout B, Buhl EH. Inhibition-based rhythms: experimental and mathematical observations on network dynamics. Int J Psychophysiology. 2000;38: 315-336.
8. Platkiewicz J, Brette R. Impact of Fast Sodium Channel Inactivation on Spike Threshold Dynamics and Synaptic Integration. PLoS Comput Biol. 2011;7: e1001129.
9. Wolfe J, Houweling AR, Brecht M. Sparse and powerful cortical spikes. Current Opinion in Neurobiology. 2010;20: 306-312.
10. Chizhov AV. Conductance-Based Refractory Density Approach: Comparison with Experimental Data and Generalization to Lognormal Distribution of Input Current. Biol Cybernetics. 2017;111(5-6): 353-364
11. Chizhov AV. Conductance-based refractory density model of primary visual cortex. J Comput Neurosci. 2014;36(2): 297-319.
12. Tsodyks M, Pawelzik K, Markram H. Neural Networks with Dynamic Synapses. Neural Computation. 1998;10(4): 821-835.
13. Loebel A, Tsodyks M. Computation by ensemble synchronization in recurrent networks with synaptic depression. J Comp Neuroscience. 2002;13: 111-124.

PDF Created with deskPDF PDF Writer - Trial :: http://www.docudesk.com
